# On the *Q* statistic with constant weights in meta-analysis of binary outcomes

**DOI:** 10.1186/s12874-023-01939-z

**Published:** 2023-06-21

**Authors:** Elena Kulinskaya, David C. Hoaglin

**Affiliations:** 1 School of Computing Sciences, University of East Anglia, Norwich Research Park, NR4 7TJ Norwich, UK; 2 Department of Population and Quantitative Health Sciences, UMass Chan Medical School, 368 Plantation Street, Worcester, Massachusetts 01605, USA

**Keywords:** Effective-sample-size weights, Inverse-variance weights, Heterogeneity, Random-effects model

## Abstract

**Background:**

Cochran’s *Q* statistic is routinely used for testing heterogeneity in meta-analysis. Its expected value (under an incorrect null distribution) is part of several popular estimators of the between-study variance, $\tau ^2$. Those applications generally do not account for use of the studies’ estimated variances in the inverse-variance weights that define *Q* (more explicitly, $Q_{IV}$). Importantly, those weights make approximating the distribution of $Q_{IV}$ rather complicated.

**Methods:**

As an alternative, we are investigating a *Q* statistic, $Q_F$, whose constant weights use only the studies’ arm-level sample sizes. For log-odds-ratio (LOR), log-relative-risk (LRR), and risk difference (RD) as the measures of effect, we study, by simulation, approximations to distributions of $Q_{IV}$ and $Q_F$, as the basis for tests of heterogeneity.

**Results:**

The results show that: for LOR and LRR, a two-moment gamma approximation to the distribution of $Q_F$ works well for small sample sizes, and an approximation based on an algorithm of Farebrother is recommended for larger sample sizes. For RD, the Farebrother approximation works very well, even for small sample sizes. For $Q_{IV}$, the standard chi-square approximation provides levels that are much too low for LOR and LRR and too high for RD. The Kulinskaya et al. (Res Synth Methods 2:254–70, 2011) approximation for RD and the Kulinskaya and Dollinger (BMC Med Res Methodol 15:49, 2015) approximation for LOR work well for $n \ge 100$ but have some convergence issues for very small sample sizes combined with small probabilities.

**Conclusions:**

The performance of the standard $\chi ^2$ approximation is inadequate for all three binary effect measures. Instead, we recommend a test of heterogeneity based on $Q_F$ and provide practical guidelines for choosing an appropriate test at the .05 level for all three effect measures.

**Supplementary Information:**

The online version contains supplementary material available at 10.1186/s12874-023-01939-z.

## Background

When the individual studies in a meta-analysis report binary outcomes in the treatment and control arms, the most common measure of effect is the odds ratio (OR) or its log (LOR). The LOR is popular in medical research, but some substantive arguments favor the relative risk or risk ratio (RR) [1]. Popular measures of effect also include the risk difference (RD).

The standard random-effects analyses routinely assess heterogeneity by using Cochran’s *Q* statistic [2], whose definition involves estimated variances of the studies’ estimated effects. To indicate the role of those variances (in the reciprocal scale), we refer to the original *Q* as $Q_{IV}$. The inverse-variance weights, based on estimated variances, underlie various shortcomings of that approach [3].

In studying estimation of the overall effect in random-effects meta-analyses of the mean difference (MD), the standardized mean difference (SMD), and LOR, we found that SSW, a weighted mean whose constant weights involve only the studies’ arm-level sample sizes, performed well, avoiding shortcomings associated with estimators that use inverse-variance weights based on estimated variances [4, 5].

We also previously studied $Q_F$, a version of Cochran’s *Q* statistic that uses those constant weights. $Q_F$ belongs to a class of generalized *Q* statistics introduced by DerSimonian and Kacker [6]. That work produced favorable results for the mean difference [7] and the standardized mean difference [8]. The present paper takes an important further step by investigating $Q_F$ for LOR, LRR, and RD.

The distribution of $Q_{IV}$ for binary effect measures is especially complex as it includes correlations between estimated weights and effects, and also depends on nuisance parameters, such as success probabilities in the control arms. The standard chi-square approximation is not reliable for small to medium sample sizes [9].

Zhang et al. [10] includes an extensive overview of statistical models for meta-analysis, summarizes the six previous studies that compared procedures for testing heterogeneity of binary-based effect measures, and, importantly, gives a comprehensive description of 30 tests of between-study heterogeneity, accompanied by simulation estimates of the Type I error rates for 29 of those tests. However, those tests do not include generalized *Q* statistics with constant weights, such as $Q_F$. In most situations they considered, Zhang et al. [10] recommend the test proposed by Kulinskaya and Dollinger [11].

In another recent publication, in the setting of sparse data under the Poisson distribution (i.e., small counts of events) Sangnawakij et al. [12] develop an exact test for heterogeneity of relative risks (i.e., ratios of event rates). Unfortunately, their simulations are limited to very sparse data (the total number of events in the two groups is 1 or 2) and appear to show deterioration in the achieved levels when the number of studies exceeds 10.

To approximate the null distribution (no heterogeneity) of $Q_F$, we derive the conditional central moments of LOR, LRR, and RD, and investigate the use of the Farebrother approximation and a two-moment gamma-approximation. Simulation of the actual null distribution of *Q* for LOR, RR, and RD enables us to study the accuracy of approximations for those null distributions and the empirical level when $\tau ^2 = 0$. For comparison we include the usual version of *Q* ($Q_{IV}$).

Section “Study-level estimation of log-odds-ratio, log-risk-ratio, and risk difference” briefly reviews study-level estimation of LOR, LRR, and RD. Section “Random-effects model and the Q statistic” reviews the generic random-effects model and describes the *Q* statistic. Section “[Sec Sec5]” discusses approximations to the distributions of $Q_F$ and $Q_{IV}$. Section “Results” describes the simulation design and summarizes the results. Section “Example: Smoking cessation” examines an example of meta-analysis using LOR and LRR. Sections “Discussion” and “Conclusions” offer a discussion and conclusions.

## Methods

### Study-level estimation of log-odds-ratio, log-risk-ratio, and risk difference

Consider *K* studies that used a particular individual-level binary outcome. Study *i* ($i = 1, \ldots , K$) reports $X_{iT}$ and $X_{iC}$, the numbers of events in the $n_{iT}$ subjects in the Treatment arm and the $n_{iC}$ subjects in the Control arm. It is customary to treat $X_{iT}$ and $X_{iC}$ as independent binomial variables:

1 $$\begin{aligned} X_{iT}\sim {\textrm{Bin}}(n_{iT},p_{iT})\qquad {\text {and}}\qquad X_{iC}\sim {\textrm{Bin}}(n_{iC},p_{iC}). \end{aligned}$$

The log-odds-ratio for Study *i* is

2 $$\begin{aligned} \theta _{i} = \log _{e} \left( \frac{p_{iT}(1 - p_{iC})}{p_{iC}(1 - p_{iT})}\right) \qquad \text {estimated by} \qquad \check{\theta }_{i} = \log _{e} \left( \frac{\check{p}_{iT}(1 - \check{p}_{iC})}{\check{p}_{iC}(1 - \check{p}_{iT})}\right) , \end{aligned}$$

where $\check{p}_{ij}$ is an estimate of $p_{ij}$.

As inputs, a two-stage meta-analysis uses estimates of the $\theta _i$ ($\hat{\theta }_i$) and estimates of their variances ($\hat{v}_i^2$). It is helpful to have an unbiased estimator of $\theta$. For a binomial random variable $X \sim {\textrm{Bin}}(n,p)$, we denote the maximum-likelihood (ML) estimator of *p* by $\tilde{p} = X/n$ . Böhning and Viwatwongkasem [13] studied estimators of *p* of the form $(X + a)/(n + 2a)$. Use of $a = 0.5$ and hence $\hat{p} = (X + 0.5)/(n + 1)$ eliminates *O*(1/*n*) bias and provides the least biased estimator of LOR [14]. We use $\hat{p}$ when estimating LOR, but also, for comparison, retain the use of $\tilde{p}$ in standard methods.

The (conditional, given the $p_{ij}$ and $n_{ij}$) asymptotic variance of $\hat{\theta }_i$, derived by the delta method, is

3 $$\begin{aligned} v_{i}^2 = \textrm{Var}(\hat{\theta }_{i}) = \frac{1}{n_{iT} {p}_{iT} (1 - {p}_{iT})} + \frac{1}{n_{iC} {p}_{iC} (1 - {p}_{iC})}, \end{aligned}$$

estimated by substituting $\hat{p}_{ij}$ for $p_{ij}$. This estimator of the variance is unbiased in large samples, but Gart et al. [14] note that it overestimates the variance for small sample sizes. They also give approximate conditional higher moments of LOR.

The log-risk-ratio (LRR) for Study *i* is

4 $$\begin{aligned} \rho _{i} = \log _{e}(p_{iT}) - \log _{e}(p_{iC}) \quad \text {estimated by} \quad \hat{\rho }_{i} = \log _{e}(\check{p}_{iT}) - \log _{e}(\check{p}_{iC}), \end{aligned}$$

where $\check{p} = (X + 1/2) / (n + 1/2)$ provides an unbiased (to order $O(n^{-2})$) estimate of $\log (p)$ [15]. An unbiased (to $O(n^{-3})$) estimate of the variance of $\hat{\rho }$ [15] is

5 $$\begin{aligned} \widehat{\textrm{Var}}(\hat{\rho }) = \frac{1}{X_T + 1/2} - \frac{1}{n_T + 1/2} + \frac{1}{X_C + 1/2} - \frac{1}{n_C + 1/2}, \end{aligned}$$

and Pettigrew et al. [15] also give approximate conditional higher moments for $\log (\hat{p})$.

The risk difference (RD) for Study *i* is

6 $$\begin{aligned} \Delta _{i} = p_{iT} - p_{iC} \quad \text {estimated by} \quad \hat{\Delta }_{i} = \tilde{p}_{iT} - \tilde{p}_{iC}. \end{aligned}$$

Its variance is

$$\textrm{Var}(\hat{\Delta }_i) = p_{iT} (1 - p_{iT}) / n_{iT} + p_{iC} (1 - p_{iC}) / n_{iC},$$

estimated by substituting $\tilde{p}$ for *p*. The moments of the binomial distribution directly yield the conditional higher moments of RD.

All three binary effect measures (LOR, LRR, and RD) have the form $\eta = h(p_T) - h(p_C)$. This relation facilitates calculation of conditional moments of $\hat{\eta }$ from the moments of *h*(*p*). Additional file 1 gives the details. Even when they are not available in closed form, our simulations yield an exact calculation of conditional central moments of $\hat{\eta }$ for all three effect measures, similar to the implementation of Kulinskaya and Dollinger [11] for LOR.

### Random-effects model and the *Q* statistic

We consider a generic random-effects model: For Study *i* ($i = 1,\ldots ,K$) the estimate of the effect is $\hat{\theta }_i \sim G(\theta _i, v_i^2)$, where the effect-measure-specific distribution *G* has mean $\theta _i$ and variance $v_i^2$, and $\theta _i \sim N(\theta , \tau ^2)$. Thus, the $\hat{\theta }_i$ are unbiased estimates of the true conditional effects $\theta _i$, and the $v_i^2 = \textrm{Var}(\hat{\theta }_i | \theta _i)$ are the true conditional variances.

Cochran’s *Q* statistic is a weighted sum of the squared deviations of the estimated effects $\hat{\theta }_i$ from their weighted mean $\bar{\theta }_w = \sum w_i\hat{\theta }_i / \sum w_i$:

7 $$\begin{aligned} Q=\sum w_i (\hat{\theta }_i - \bar{\theta }_w)^2. \end{aligned}$$

In [2] $w_i = 1/\hat{v}_i^2$, the reciprocal of the *estimated* variance of $\hat{\theta }_i$, hence the notation $Q_{IV}$. In what follows, we examine $Q_F$, discussed by DerSimonian and Kacker [6] and further studied by Kulinskaya et al. [7], in which the $w_i$ are arbitrary positive constants. In $Q_F$ we specify $w_i = \tilde{n}_i = n_{iC} n_{iT} / n_i$, the effective sample size in Study *i* ($n_i = n_{iC} + n_{iT}$).

Define $W = \sum w_i$, $q_i = w_i / W$, and $\Theta _i = \hat{\theta }_i - \theta$. In this notation, and expanding $\bar{\theta }_w$, Eq. (7) can be written as

8 $$\begin{aligned} Q = W \left[ \sum q_i (1 - q_i) \Theta _i^2 - \sum \limits _{i \not = j} q_i q_j \Theta _i \Theta _j \right] . \end{aligned}$$

We distinguish between the conditional distribution of *Q* (given the $\theta _i$) and the unconditional distribution, and the corresponding moments of $\Theta _i$. For instance, the conditional second moment of $\Theta _i$ is $M_{2i}^c = v_i^2$, and the unconditional second moment is $M_{2i} = \textrm{E}(\Theta _i^2) = \textrm{Var}(\hat{\theta }_i) = \textrm{E}(v_i^2) + \tau ^2$.

Under the generic REM, it is straightforward to obtain the first moment of $Q_F$ as

9 $$\begin{aligned} \textrm{E}(Q_F) = W \left[ \sum q_i (1 - q_i) \textrm{Var}(\Theta _i) \right] = W \left[ \sum q_i (1 - q_i) (\textrm{E}(v_i^2) + \tau ^2) \right] . \end{aligned}$$

This expression is similar to Eq. (4) in [6]; DerSimonian and Kacker use $v_i^2$ instead of its unconditional mean ${\textrm{E}}(v_i^2)$.

Kulinskaya et al. [7] also provide expressions for the second and third moments of $Q_F$, but these moments require higher moments of $\Theta$, up to the fourth and the sixth moments, respectively. The variance of *Q* is given by

10 $$\begin{aligned} W^{-2} \textrm{Var}(Q) = \sum \limits _i q_i^2 (1-q_i)^2 (M_{4i} - M_{2i}^2) + 2 \sum \limits _{i \not = j} q_i^2 q_j^2 M_{2i} M_{2j}, \end{aligned}$$

where $M_{4i} = {\textrm{E}}(\Theta _i^4)$ is the fourth (unconditional) central moment of $\hat{\theta }_i$.

Section "Study-level estimation of log-odds-ratio, log-risk-ratio, and risk difference" discusses obtaining the conditional moments of LOR, LRR, and RD. However, the unconditional moments depend on the mechanism generating the arm probabilities $p_{iC}$ and $p_{iT}$.

In the standard REM for LOR, we would assume ${\textrm{logit}}(p_{iT}) = {\textrm{logit}}(p_{iC}) + \theta _i$ for $\theta _i \sim N(\theta ,\tau ^2)$. The intercept $p_{iC}$ may also be random (i.e., $p_{iC}\sim H(\cdot )$). Further, $p_{iC}$ and $p_{iT}$ may be correlated. Similarly, in the standard REM for RD, we would assume $p_{iT} = p_{iC} + \Delta _i$ for $\Delta _i \sim N(\Delta ,\tau ^2)$. However, the distribution of $\Delta _i$ needs to be restricted, to ensure that probabilities lie within (0,1). Before this is resolved, we cannot derive unconditional moments of RD. Similarly, for LRR, we assume that $p_{iT} = \exp (\log p_{iC} + \rho _i)$, and the distribution of $\rho _i$ needs to be restricted to keep values of $p_{iT}$ within (0,1). Bakbergenuly et al. [16] give a detailed discussion.

Fortunately, in the fixed-intercept models (i.e., when the $p_{iC}$ are fixed), assuming also homogeneity of effects ($\tau ^2 = 0$), the unconditional and conditional moments of each binary effect measure coincide. Therefore, the conditional moments of *Q* are sufficient to obtain a moment-based approximation to the distribution of $Q_F$ under homogeneity.

### Approximations to the null distributions of $Q_F$ and $Q_{IV}$

For meta-analysis of mean differences (MD), Kulinskaya et al. [7] considered the distribution of $Q_F$, a quadratic form in normal variables, which has the form $Q = \Theta ^{T}A\Theta$ for a symmetric matrix *A* of rank $K - 1$. Because, for MD, the vector $\Theta$ has a multivariate normal distribution, $N(\mu ,\Sigma )$, the distribution of $Q_F$ can be evaluated by the algorithm of Farebrother [17] (after determining the eigenvalues of $A \Sigma$ and some other inputs). If the variances in $\Sigma$ are the true variances, Farebrother’s algorithm evaluates the exact distribution of *Q*. In practice (as in our simulations), it is necessary to plug in estimated variances. Encouragingly, the resulting approximation is quite accurate for MD. Kulinskaya et al. [7] also considered a two-moment approximation and a three-moment approximation. The three-moment approximation regularly encountered numerical problems, so we do not include it here.

For the binary effect measures, $Q_F$ is a quadratic form in asymptotically normal variables. The Farebrother algorithm may provide a satisfactory approximation for larger sample sizes, though it may not behave well for small *n*. To apply it, we again plug in conditional or unconditional estimated variances. (In the fixed-intercept models, the two coincide under the null, $\tau ^2 = 0$.) We investigate the quality of that approximation, which we denote by F SSW, and the two-moment approximation (2M SSW), which is based on the gamma distribution. For each of these two approximations, we investigate two approaches to estimating $p_{iT}$ to plug into the calculation of the second and fourth central moments of an effect measure, $\hat{\eta }_i$. The “naïve” approach estimates $p_{iT}$ from $X_{iT}$ and $n_{iT}$. For the “model-based” approach, we observe that each of LOR, LRR, and RD has the form $\eta = h(p_T) - h(p_C)$, which facilitates calculation of conditional moments of $\hat{\eta }$ from the moments of *h*(*p*). We obtain estimated moments from the relation $\widehat{h(p_{iT})} = \widehat{h(p_{iC})} + \bar{\eta }$ for a fixed-weights mean effect $\bar{\eta }$. Thus, we study four new approximations to the null distribution of $Q_F$: F SSW naïve, F SSW model, 2M SSW naïve, and 2M SSW model.

The null distribution of $Q_{IV}$ is usually approximated by the chi-square distribution with $K - 1$ degrees of freedom. For the binary effect measures, as also for both MD and SMD, this approximation is not accurate for small sample sizes [9]. For RD and LOR, Kulinskaya et al. [18] and Kulinskaya and Dollinger [11], respectively, provided an improved approximation to the null distribution of $Q_{IV}$ based on a two-moment gamma approximation; we denote the approximation for LOR by KD and that for RD by KDB. Biggerstaff and Jackson [19] used the Farebrother approximation to the distribution of a quadratic form in normal variables as the “exact” distribution of $Q_{IV}$. Jackson et al. [20] extended this approach to a *Q* with arbitrary weights in a meta-regression setting. When $\tau ^2 = 0$, the Biggerstaff and Jackson [19] approximation to the distribution of $Q_{IV}$ is the $\chi ^2_{K - 1}$ distribution.

## Results

### Simulation design

Our simulation design follows that described in [5]. Mainly, we varied four parameters: the overall true effect ($\theta$, $\rho$, or $\Delta$), the number of studies (*K*), the studies’ total sample size (*n* or $\bar{n}$, the average sample size), and the probability in the control arm ($p_{iC}$). We kept the proportion of observations in the control arm (*f*) at 1/2. We generated only the null distribution of *Q* (the between-studies variance $\tau ^2 = 0$). To study power of the test for heterogeneity of LOR, however, we also varied the between-studies variance ($\tau ^2$).

For LOR the values of $\theta$ (0, 0.1, 0.5, 1, 1.5, and 2) aim to represent the range containing most values encountered in practice. LOR is a symmetric effect measure, so positive values of $\theta$ suffice. However, for RR we considered both negative and positive values of $\rho$ from $-1.5$ to 1.5 in steps of 0.5. For RD, for comparative purposes, we used the same pairs $(p_{iC}, p_{iT})$ as for RR. Table 1 gives the details.

The numbers of studies (*K* = 5, 10, and 30) reflect the sizes of many meta-analyses and have yielded valuable insights in previous work.

The values of $\tau ^2$ (0(0.1)1) systematically cover a reasonable range.

In practice, many studies’ total sample sizes fall in the ranges covered by our choices (*n* = 20, 40, 100, and 250 when all studies have the same *n*, and $\bar{n}$ = 30, 60, 100, and 160 when sample sizes vary among studies). The choices of sample sizes corresponding to $\bar{n}$ follow a suggestion of Sánchez-Meca and Marín-Martínez [21], who constructed the studies’ sample sizes to have skewness 1.464, which they regarded as typical in behavioral and health sciences. For $K = 5$, Table 1 lists the sets of five sample sizes. The simulations for $K = 10$ and $K = 30$ used each set of unequal sample sizes twice and six times, respectively.

The values of $p_{iC}$ are .1, .2, and .5 to provide a typical range of small to medium risks.

The values of $p_{iC}$ and true effect ($\theta _i$, $\rho _i$ or $\Delta _i$) defined the probabilities $p_{iT}$, and the counts $X_{iC}$ and $X_{iT}$ were generated from the respective binomial distributions. We used a total of 10, 000 repetitions for each combination of parameters. We discarded “double-zero” and “double-*n*” studies and reduced the observed value of *K* accordingly. Next, we discarded repetitions with $K<3$ and used the observed number of repetitions for analysis.

R statistical software [22] was used for simulations. The user-friendly R programs implementing our methods are available in [23], and we report the detailed simulation results in [24].

**Table 1:** Values of parameters in the simulations

Parameter	Equal study sizes	Unequal study sizes
*K* (number of studies)	5, 10, 30	
*n* or $\bar{n}$ (average (individual) study size —	20, 40, 100, 250	30 (12,16,18,20,84),
total of the two arms)		60 (24,32,36,40,168),
For $K = 10$ and $K = 30$, the same set of unequal		100 (64,72,76,80,208),
study sizes is used twice or six times, respectively.		160 (124,132,136,140,268)
*f* (proportion of observations in the control arm)	1/2	
$p_{iC}$ (probability in the control arm)	.1, .2, .5	
$\theta$ (true value of LOR)	0, 0.1, 0.5, 1, 1.5, 2	
$\tau ^{2}$ (variance of random effects for LOR)	0(0.1)1	
$\rho$ (fixed value of LRR)		
For $p_{iC} = .1$ or .2	$-0.5$, 0, 0.5, 1, 1.5	
For $p_{iC} = .5$	$-1.5$, $-1$, $-0.5$, 0, 0.5	
$p_T$ (fixed probability in the treatment arm)		
for RD (and for RR),		
when $p_{iC} = .1$	.06, .10, .16, .27, .45	
when $p_{iC} = .2$	.12, .20, .33, .54, .90	
when $p_{iC} = .5$	.11, .18, .30, .50, .82	

### Evaluating the approximations to the null distributions of $Q_F$ and $Q_{IV}$

Under the null hypothesis the *p*-values of a parametric test, obtained from the (continuous) distribution function of the test statistic, are uniformly distributed on $[0,\;1]$. Our simulations produce information on the accuracy of an approximation, $\hat{F}$, for the distribution function of *Q*. From the value of *Q* in each of *M* iterations, we calculate the *p*-value, $\tilde{p} = 1 - \hat{F}(Q)$. For selected values of the upper tail area *p*, the results of the *M* iterations yield $\hat{p}(\hat{F},p) = \#(\tilde{p} < p)/M$, which estimates $P(1 - \hat{F}(Q) < p)$, the actual level (or rejection rate) of the approximate test based on *Q*, at nominal level *p*. Conveniently, this approach does not require the true distribution function of *Q*, which is generally not available in closed form.

We can examine these results by plotting $\hat{p}(\hat{F},p)$ versus *p*, a type of probability–probability (P–P) plot [25]. To focus on the difference, we flatten the P–P plot by plotting the error, $\hat{p}(\hat{F},p) - p$ versus *p*. The importance of a given error varies with *p* (e.g., the error cannot be more negative than $-p$), so a further step plots the relative error, $(\hat{p}(\hat{F},p) - p) / p$ versus *p*. Because of the use of the values of $\tilde{p}$ in assessing heterogeneity, we judge the performance of the approximations by their relative errors when *p* is in the usual range, say from .01 to .1. The figures for relative error in the next sections go a little farther, to $p = .25$.

To show the performance of various tests of heterogeneity, we plot achieved empirical levels of the corresponding approximations at the nominal .05 level versus $\theta$, $\rho$, and $\Delta$, respectively. The figures show $0 \le \theta \le 2$ for LOR, $-0.5 \le \rho \le 1.5$ for LRR, and $-0.04 \le \Delta \le 0.35$ for RD. For LOR, we also plot empirical power of the tests based on the approximations under study at the nominal .05 level versus $\tau ^2$.

**Fig. 1:**
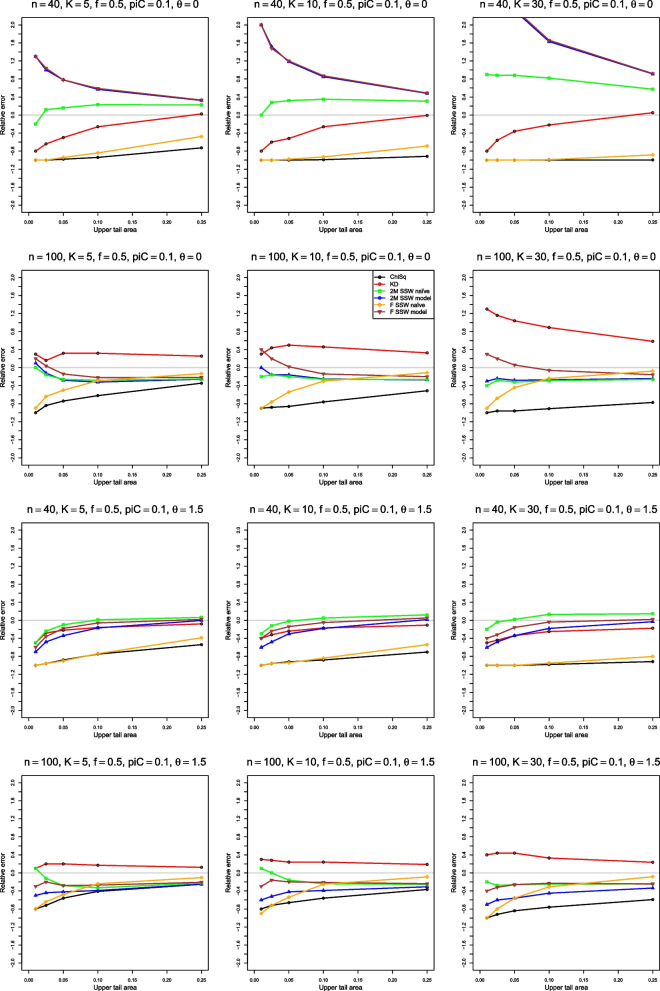
Relative error in the level of the test for heterogeneity of log-odds-ratio, vs upper tail area, for four approximations to the null distribution of $Q_F$ and two approximations to the null distribution of $Q_{IV}$, when $p_{iC} = .1$. The rows correspond to the combinations of $\theta = 0$ and $\theta = 1.5$ with $n = 40$ and $n = 100$

**Fig. 2:**
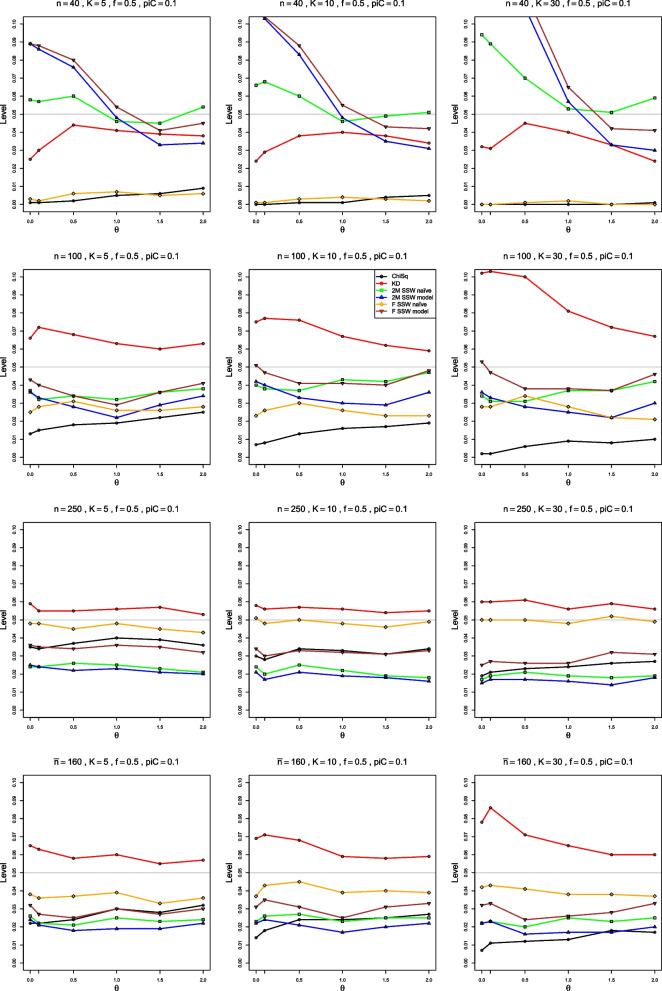
Empirical levels at nominal level of significance .05, vs $\theta$, for four approximations to the null distribution of $Q_F$ for log-odds-ratio and for two approximations to the null distribution of $Q_{IV}$, when $p_{iC} = .1$. First three rows: equal sample sizes $n = 40,\;100$ and 250; fourth row: unequal sample sizes, $\bar{n}=160$

**Fig. 3:**
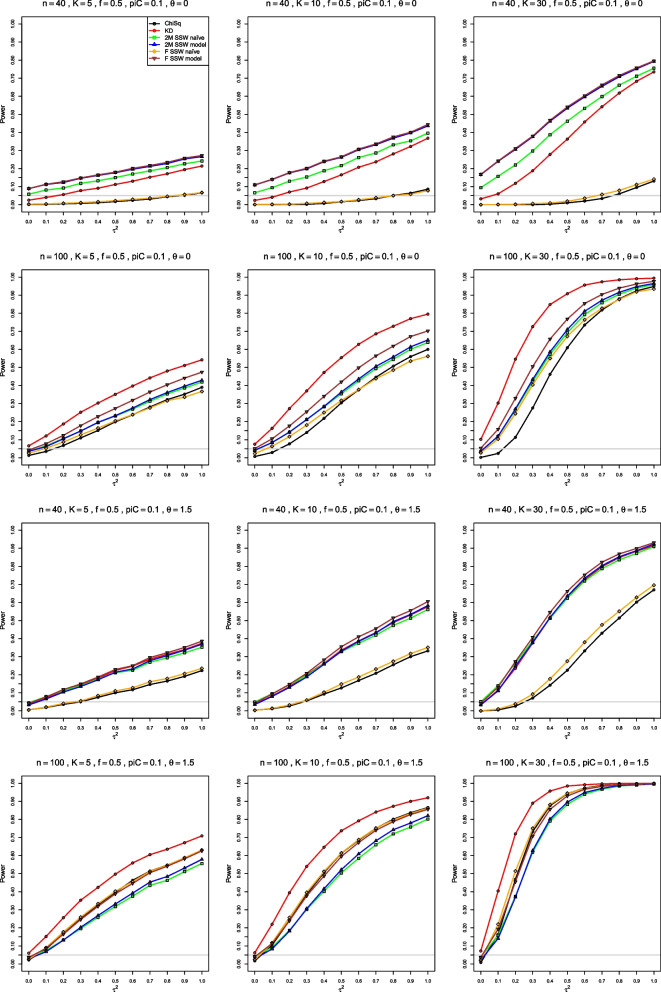
Empirical power at nominal level $\alpha = .05$ vs $\tau ^2$, for four approximations to the distribution of $Q_F$ for log-odds-ratio and for two approximations to the null distribution of $Q_{IV}$, when $p_{iC} = .1$, $f = .5$, equal sample sizes. The rows correspond to the combinations of $\theta = 0$ and $\theta = 1.5$ with $n = 40$ and $n = 100$

**Fig. 4:**
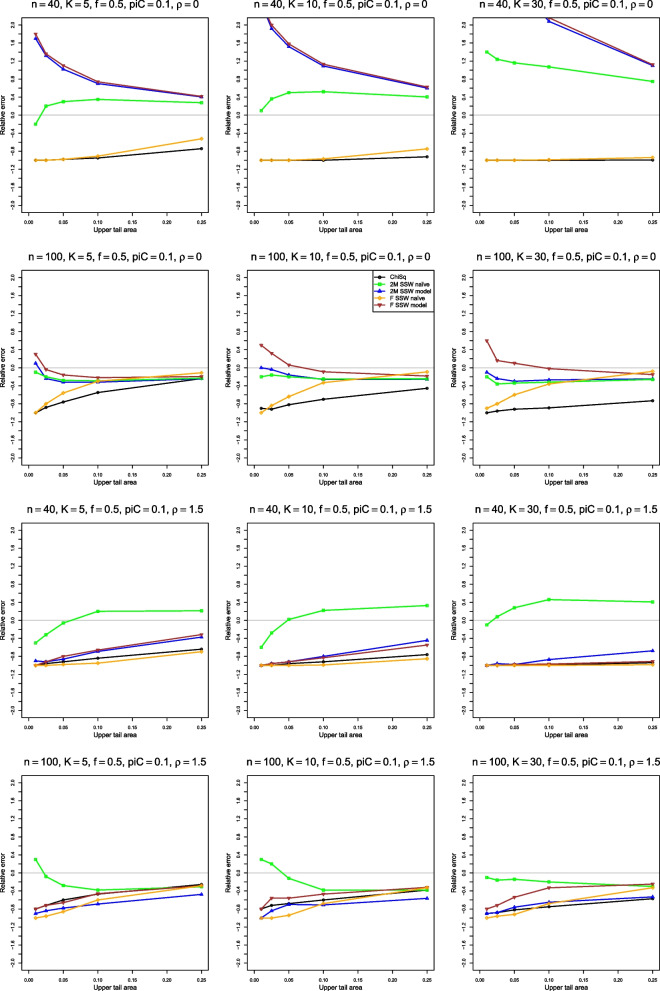
Relative error in the level of the test for heterogeneity of log-relative-risk, vs upper tail area, for four approximations to the null distribution of $Q_F$ and the chi-square approximation to the null distribution of $Q_{IV}$, when $p_{iC} = .1$. The rows correspond to the combinations of $\rho = 0$ and $\rho = 1.5$ with $n = 40$ and $n = 100$

**Fig. 5:**
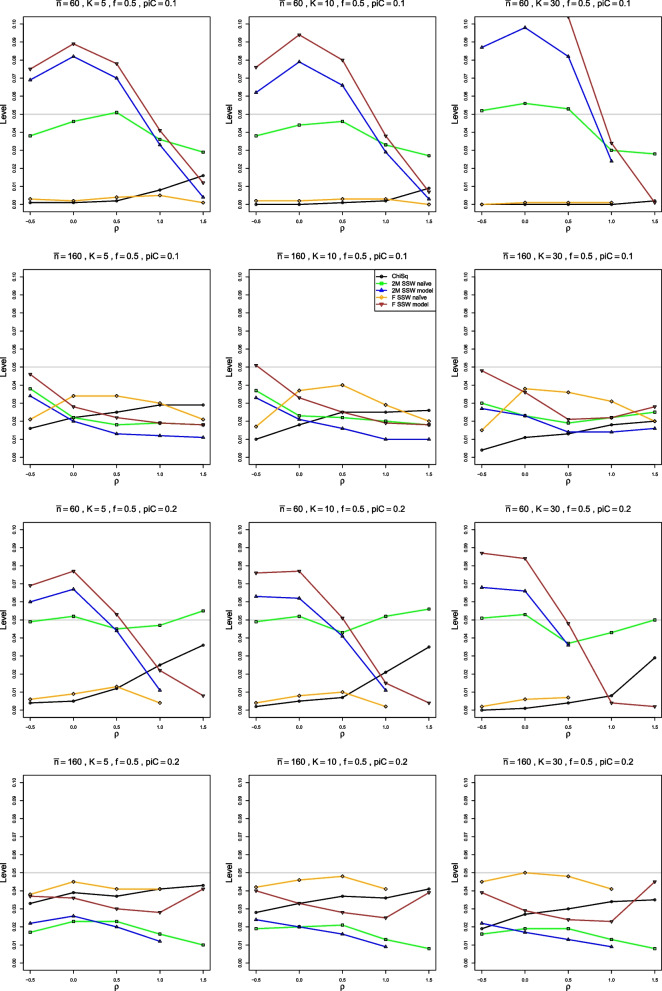
Empirical level at nominal level of significance .05, vs $\rho$, for four approximations to the null distribution of $Q_F$ for log-relative-risk and for the standard chi-square approximation to the null distribution of $Q_{IV}$, for unequal sample sizes, $\bar{n} = 60$ and 160. First two rows: $p_{iC} = .1$; second two rows: $p_{iC} = .2$

**Fig. 6:**
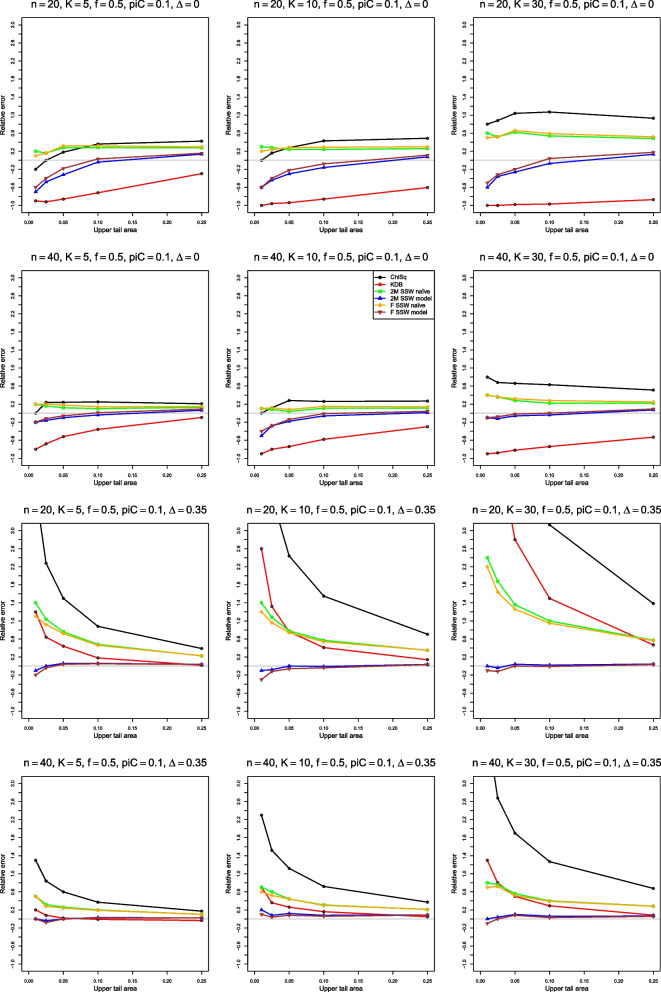
Relative error in the level of the test for heterogeneity of risk difference, vs upper tail area, for four approximations to the null distribution of $Q_F$ and two approximations to the null distribution of $Q_{IV}$, when $p_{iC} = .1$. The rows correspond to the combinations of $\Delta = 0$ and $\Delta = 0.35$ with $n = 20$ and $n = 40$

**Fig. 7:**
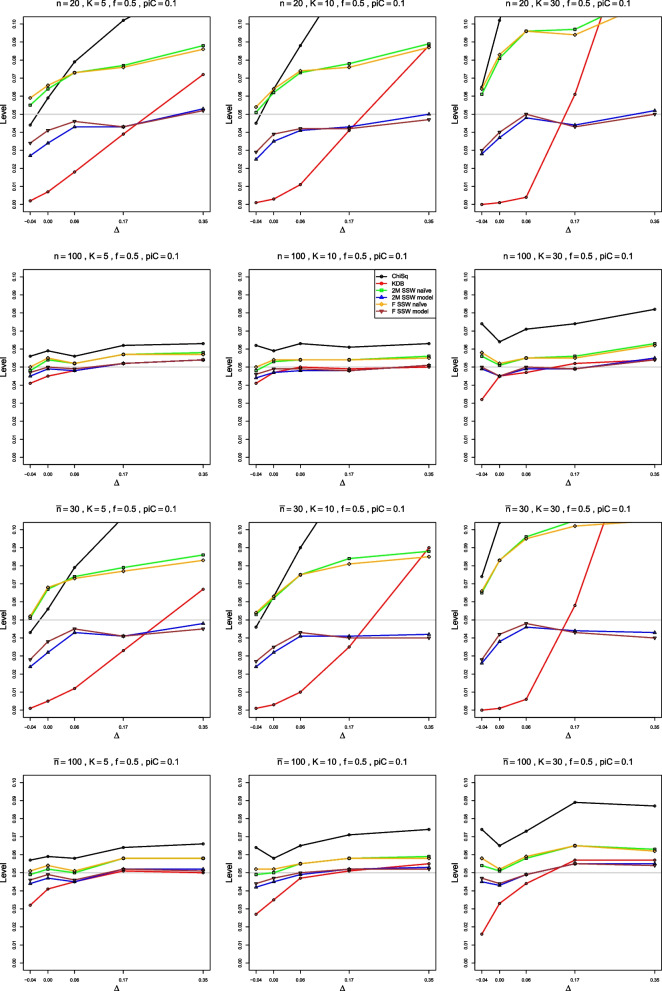
Empirical level at nominal level of significance .05, vs $\Delta$, for four approximations to the null distribution of $Q_F$ for risk difference and for two approximations to the null distribution of $Q_{IV}$, when $p_{iC} = .1$. First two rows: equal sample sizes $n = 40$ and 100; second two rows: unequal sample sizes, $\bar{n} = 30$ and 100

Figures 1, 2, 3, 4, 5, 6 and 7 are based on $p_{iC} = .1$. Additional figures for $p_{iC} = .2$ and $p_{iC} = .5$ are in Additional file 1.

Our full simulation results are available as an arXiv e-print [24]. The figures in the e-print include flattened P–P plots for *p* = .001, .0025, .005, .01, .025, .05, .1, .25, .5 and the complementary values .75, ..., .999, and plots at empirical level .05, but not relative error plots.

Although the summaries do not separate $Q_F$ and $Q_{IV}$, the two 2M SSW approximations and the two F SSW approximations pertain to the null distribution of $Q_F$, and ChiSq, KD, and KDB pertain to the null distribution of $Q_{IV}$.

### Simulation results for approximations to the null distributions of $Q_F$ and $Q_{IV}$ for LOR

#### Relative error

None of the six approximations has smaller relative error than any of the others. ChiSq, however, often has the largest relative error (in magnitude), at values of *p* that matter in practice. Also, situations with $K = 30$ and small *n* (or $\bar{n}$) are especially challenging for all six approximations.

Figure 1 shows relative error when $p_{iC} = .1$. For $\theta \le 0.5$, 2M SSW naïve has relative error closest to 0 when $n \le 40$ (KD is second best), and 2M SSW model and F SSW model are the best approximations when $n \ge 100$. However, when $\theta \ge 1$, ChiSq is always the worst, and the performance of the other approximations varies with *n* and $\theta$. For example, F SSW naïve is no better than ChiSq when $n \le 40$, is slightly better when $n = 100$, and has very little error when $n = 250$; KD has substantial negative error when $n = 20$, is inferior to 2M SSW naïve when $n = 40$, and has small positive error (increasing with *K*) when $n = 100$. As a single choice when $\theta \ge 1$, 2M SSW naïve seems satisfactory.

When $p_{iC} = .2$, 2M SSW naïve works well for $n \le 40$, but F SSW naïve is best when $n \ge 100$ (Fig. A.1).

When $p_{iC} = .5$, F SSW naïve is the best approximation for $n \le 100$ when $\theta \le 1$. For $\theta \ge 1.5$, F SSW model works better than F SSW naïve for $n<100$. (Figure A.2). Results are similar for equal and unequal sample sizes.

In the above summary, the dependence on $p_{iC}$ and $\theta$ shows the challenge of choosing an approximation for the null distribution of *Q* for LOR. We can readily exclude ChiSq, which never fits the distribution of $Q_{IV}$ well. Otherwise, the best approach is unclear.

#### Empirical level

When $p_{iC} = .1$, all methods are rather erratic for very small sample sizes. But, for equal sample sizes as small as 40, 2M SSW naïve has reasonable empirical levels, from .045 to .07 when $K\le 10$. For $K = 30$, its levels are too high for $\theta < 0.5$, but close to the nominal .05 thereafter (Fig. 2). 2M SSW naïve also performs well for unequal sample sizes when $\bar{n} = 60$ (not shown). When $n \ge 100$, F SSW model performs as well as or better than (for larger *K*) 2M SSW naïve, with levels for both methods somewhat lower than nominal. KD also has reasonable levels, somewhat higher than nominal, when $K = 5$ and 10. F SSW naïve and ChiSq both have very low levels for $n < 100$, but when $n = 250$, F SSW naïve achieves practically nominal levels. KD is then the second best, with levels between .05 and .06, and levels of all other methods are too low. The behavior is similar for unequal sample sizes, as Fig. 2 shows for $\bar{n} = 160$.

When $p_{iC} = .2$, 2M SSW naïve and F SSW model (for $\theta < 0.5$) are the best choices for small sample sizes such as $\bar{n} = 30$ and $n = 40$, and F SSW naïve is the best choice for $n \ge 100$ or $\bar{n} \ge 100$, achieving nominal levels (Fig. A.3). KD is second best, with levels that are somewhat too high, and all other methods have very low empirical levels.

When $p_{iC} = .5$, F SSW model performs very well for very small sample sizes, $n=20$ and $\bar{n}=30$. F SSW naïve and KD are the best choices for $n \ge 100$, though the former’s levels are somewhat too low when $\theta = 2$ (Fig. A.4).

In summary, 2M SSW naïve is the best choice for small sample sizes, and F SSW naïve works well for large sample sizes, when LOR is approximately normal. This is achieved when $n \ge 100$ for $p_{iC} \ge .2$ and only at $n = 250$ for $p_{iC} = .1$. KD is also a good choice when $n \ge 100$.

#### Empirical power

Proper comparisons of power assume that the tests have the same level (rejection rate under the null hypothesis). We have not attempted to modify the approximations, to align their levels with the nominal .05 level. Thus, our simulations yielded empirical power at the nominal .05 level.

All methods have rather low empirical power when $n \le 40$. It increases with *n*, *K*, $\theta$, and $p_{iC}$. *K* has the strongest impact, followed by $p_{iC}$, $\theta$, and *n*. The order of the actual levels for all tests mostly defines the order of their power at all $\tau ^2$ values. The only exception is that the power curves of ChiSq and F SSW naïve sometimes cross (Figs. 3, A.5, A.6).

ChiSq has the lowest empirical power. For example, when $p_{iC} = .1$, $\theta = 0$, and $n = 40$, its power is less than .05 for $0 \le \tau ^2 \le 0.7$. Depending on *n* and $\theta$, either 2M SSW naïve or F SSW naïve is usually the second worst, probably because their actual levels are too low. All three have extremely low power when $n \le 40$. KD also has very low power when $n=20$, but its power improves, starting at $n = 40$; it often has the highest power when $n \ge 100$, but its levels are also too high then.

Overall, when $p_{iC}=.1$, power is reasonable when $n \ge 100$ and $K = 30$, Fig. 3, or when $n = 250$ for smaller *K*. For $p_{iC}\ge .2$, power improves when $n \ge 40$, and all methods have similar power when $n \ge 100$ (Figs. A.5, A.6).

### Simulation results for approximations to the null distributions of $Q_F$ and $Q_{IV}$ for LRR

2M SSW model and F SSW naïve often failed to converge for large values of $\rho$, especially for small *n*. As an example, for $p_{iC} = .1$, $K = 30$, and $\rho = 1.5$, the number of repetitions that converged was only 716 when $n = 20$ and only 1890 when $n = 40$. Similarly, even more problematic combinations of parameters were $p_{iC} = .2$, $\rho =1.5$, $K = 5, 10$ and 30 and $p_{iC} = .5$, $\theta = 0.5$, $K = 5, 10$ and 30, where only a few repetitions converged. For some of these combinations (but not for others), $p_{iT}$ was rather high, at .82 or .9, perhaps resulting in removal of more “double-*n*” studies. In our plots, we discarded all combinations that had less than 20% convergence.

#### Relative error

Figure 4 shows that, when $p_{iC} = 0.1$, four of the five approximations are completely unsatisfactory for very small sample sizes. 2M SSW naïve is better than the others for *p* between .01 and .1, but far from usable. For $n \ge 100$, F SSW model, 2M SSW model, and 2M SSW naïve usually provide a reasonable fit when $\rho \le 1$, and 2M SSW naïve is better than the other two for larger $\rho$. Overall, the quality of all approximations deteriorates as *K* increases.

When $p_{iC} = 0.2$, 2M SSW naïve is best for $n = 20$, and both 2M approximations work well for $n = 40$. For $n \ge 100$, F SSW naïve is the best when $\rho \le 1$, but ChiSq is the best when $\rho = 1.5$ (Fig. A.7).

When $p_{iC} = .5$, 2M SSW naïve is a good choice for small *n* when $\rho \le 0$, but F SSW model appears to fit better when $\rho > 0$ or $n \ge 100$. Uncharacteristically, ChiSq is also not a bad choice when $p_{iC} = .5$ and $\rho > 0$ (Fig. A.8).

#### Empirical level

For very small sample sizes and $p_{iC} = .1$, no tests are reliable. 2M SSW naïve is the best choice from $n = 40$ or $\bar{n} = 30$ to $n = 100$ (Figs. 5 and A.9), though when $n = 40$ it behaves erratically for $K = 30$ (Fig. A.9). For these sample sizes, the levels of 2M SSW naïve are often below nominal, though they typically stay above .03. For larger sample sizes, the levels become too low, and we do not recommend 2M SSW naïve then. F SSW model works well when $K = 5$ and $n = 100$ for equal, but not for unequal, sample sizes.

ChiSq and F SSW naïve have extremely low levels, near zero, for $n \le 100$. The levels improve for larger sample sizes, with F SSW naïve approaching nominal levels faster. By $\bar{n} = 160$, this is the best method when $\rho \ge 0$, and the levels are around .03 at .05 nominal. By $n = 250$, F SSW naïve holds the nominal .05 level well. For $\rho < 0$ (i.e., for small probabilities in both arms), F SSW model works well when $\bar{n} = 160$. For larger $\rho$ and $K=5$ or 10, ChiSq seems better than F SSW model.

When $p_{iC} = .2$, 2M SSW naïve is the best approximation for $n < 100$, and F SSW naïve for $n \ge 100$ (Figs. 5 and A.9).

When $p_{iC} = .5$, F SSW model is the best method in all cases (Fig. A.10).

In summary, we recommend 2M SSW naïve for $n < 100$ and F SSW naïve for larger sample sizes when $p_{iC} \le .2$. The choice between the two methods for $n = 100$ depends on the value of $p_{iC}$: 2M SSW naïve when $p_{iC} = .1$, F SSW naïve when $p_{iC} = .2$. When $p_{iC} = .5$, F SSW model is the best choice.

### Simulation results for approximations to the null distributions of $Q_F$ and $Q_{IV}$ for RD

Only KDB had convergence issues for very small sample sizes combined with small probabilities. The worst convergence, only 35.3%, occurred for $p_{iC} = .1$, $\Delta = -0.04$ and $K = 5$. For $n = 40$, the same configuration resulted in 83.8% convergence. The only other problematic configuration was $p_{iC} = .2$, $\Delta = -0.08$, $n = 20$ and $K = 5$, with a convergence rate of 86.1%.

#### Relative error

Figure 6 shows that the 2M SSW model and F SSW model approximations fit well when $p_{iC} = .1$, starting from $n = 20$.

When $p_{iC} = .2$ (Fig. A.11), 2M SSW model and F SSW model work well for small sample sizes, and so does KDB unless $\Delta \ge 0.7$, making $p_{iT} \ge .9$. In that case, both SSW naïve approximations work well.

When $p_{iC} = .5$ (Fig. A.12), 2M SSW model and F SSW model work well for $n \ge 20$. All approximations fit reasonably well for $n \ge 100$.

#### Empirical level

Empirical levels of the F SSW model approximation to the null distribution of $Q_F$ are reasonably close to the nominal .05 level for equal sample sizes as low as $n = 20$ and unequal sample sizes as low as $\bar{n} = 30$, and the 2M SSW model approximation is almost as good. For $p_{iC} = .1$, the empirical levels are about .03 at $\Delta = -0.04$ but increase to .04 or better for non-negative values of $\Delta$. When $n \ge 100$ or $\bar{n} \ge 100$, both approximations are very close to the nominal level. The KDB approximation is much too conservative for small sample sizes, but by $n = 100$ it has levels between .04 and .05 for $\Delta \ge 0$. The naïve approximations to the distribution of $Q_F$ have levels that are too high for $\Delta \ge 0$ and decrease slowly with *n*, to between .05 and .06 at $n = 100$. ChiSq has even higher levels, especially for large values of $\Delta$ at $K = 30$. When $n = 100$, its levels are between .07 and .09 at $K = 30$ (Fig. 7).

For $p_{iC} = .2$, the results are similar. The two model-based approximations provide very good results by $n = 40$ or $\bar{n} = 60$, KDB performs somewhat better, and, for $K = 30$ and large $\Delta$, ChiSq is even worse (Fig. A.13). The results are also similar for $p_{iC} = .5$. The main difference is that achieved empirical levels are relatively insensitive to increase in $\Delta$. Thus, for $n = 100$, ChiSq provides stable levels of about .06 for $K = 5$, .07 for $K = 10$ and .08 for $K = 30$ (Fig. A.14).

In summary, we recommend using F SSW model, which provides very good results for very small sample sizes, $n = 20$ or $\bar{n} = 30$.

### Example: Smoking cessation

**Fig. 8:**
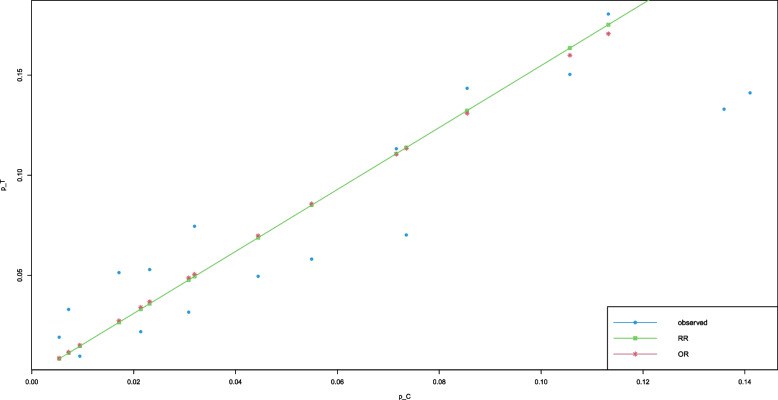
Observed values of $\hat{p}_{iT}$ vs $\hat{p}_{iC}$ (blue dots) for the original 17 studies in the meta-analysis by Stead et al. [26] of single-visit interventions for smoking cessation, and their expected values obtained by the IV MP method. Green: based on LRR $\hat{\rho }=0.4369$; red: based on LOR $\hat{\theta }= 0.4774$

Stead et al. [26] conducted a systematic review of clinical trials on the use of physician advice for smoking cessation. We use the data from the subgroup of interventions in which the treatment involved only one visit (Comparison 3.1.4, p. 54). The first version of the report was published in 2001. In an update, published in 2004, 17 studies included this comparison. The 2013 update includes one more study, by Unrod (2007). For each study, Table A.1 gives the number of subjects in the Treatment and Control arms and the number who were nonsmokers at the longest follow-up time reported (either 6 months or 12 months). The definition of “nonsmoker” varies among the studies. Some studies required sustained abstinence, and others only asked about smoking status at that time (point prevalence). Stead et al. analyzed relative risk. We analyze both odds ratio and relative risk. Kulinskaya and Dollinger [11] analyzed the odds ratio. Figure 8 shows that both OR and RR are reasonable effect measures for these data.

One feature of the figure stands out: the single-visit interventions were not a great success. No study’s cessation rate exceeded 20%. Interestingly, of the seven studies with cessation rates above 10% in the Treatment arm, all reported a point prevalence based on subjects’ self-reports, and only two of them used any validation (e.g., expired CO or salivary cotinine). (Two studies reported relatively low point prevalence, and one of those used a form of validation.) The weaker the outcome measure, the easier it is to achieve success.

**Table 2:** The *p*-values from six methods for testing heterogeneity in the meta-analysis by Stead et al. on the use of physician advice for smoking cessation

Data	Measure	$\chi ^2$	KD	2M SSW model	2M SSW naïve	F SSW model	F SSW naïve
17 studies	LOR	0.079	0.035	0.088	0.085	0.050	0.038
	LRR	0.059		0.081	0.078	0.044	0.032
18 studies	LOR	0.107	0.052	0.090	0.087	0.052	0.038
	LRR	0.080		0.082	0.079	0.046	0.033

The studies were mostly balanced, though two studies had substantially more subjects in the treatment arm. Sample sizes varied from 182 to 3128, with an average of 836 patients per study. The mean probabilities of smoking cessation were rather low in both arms, at .058 in the treatment arm and .043 in the control arm.

The standard LOR IV-based meta-analysis of the original 17 studies by the method of Mandel and Paule (MP) [27] used by Stead et al. gives $\hat{\theta }= 0.4774$ with standard error 0.1148 and $p < .0001$ for the intervention effect ($\hat{\tau }^2_{MP}=0.0754$). The fixed-weights effect estimate of LOR is higher, at 0.7127. $Q_{IV} = 24.84$, and the chi-square approximation on 16 df provides a *p*-value of .079. Table 2 gives the *p*-values for all methods of testing heterogeneity. As the sample sizes are rather high, our simulations suggest that the KD and F SSW model methods give *p*-values closest to nominal (Fig. 2, row 4). These two *p*-values are .035 and .05, respectively. F SSW naïve gives a similar *p*-value of .38. The other three methods provide considerably higher *p*-values.

The results for LRR in these 17 studies are similar. The standard MP IV-based meta-analysis for LRR results in $\hat{\rho }= 0.4369$ with standard error 0.1105 and $p < .0001$ for the intervention effect ($\hat{\tau }^2_{MP} = 0.0759$). The fixed-weights effect estimate of LRR is higher, at $\hat{\rho } = 0.6883$. The $Q_{IV}$ statistic is 25.69, with a chi-square-based *p*-value of .059. Table 2 shows the *p*-values for all methods. From our simulation results (Fig. 5, row 4), we expect the F SSW naïve method to have *p*-value closest to nominal at .032, and the rest of the methods to give somewhat higher *p*-values. This is exactly what happens in this example.

In the analysis of LOR, addition of the 18th study somewhat increased the *p*-value for the standard *Q* test, to .107, and the KD *p*-value to .052, but it hardly affected the *p*-values of the four new methods. The recommended F SSW naïve test rejects homogeneity of LRR at the .05 significance level, with p = .033.

## Discussion

Cochran’s *Q* statistic [2] is widely used for testing heterogeneity in meta-analysis. It also underlies a number of popular estimators of the heterogeneity variance $\tau ^2$. Therefore, reliable approximations for its null distribution are important to the practice of meta-analysis. However, its use of inverse-variance weights based on estimated variances and the complicated relation between effect measures and those weights make it difficult to obtain good approximations to the null distribution of $Q_{IV}$. Our previous work for the mean difference and the standardized mean difference [7, 8] considered, instead, approximations to the null distribution of the alternative statistic $Q_F$, which uses weights based on effective sample sizes. For both effect measures, the Farebrother approximation worked well.

In the present study, we proposed four new approximations to the null distribution of $Q_F$ for the three binary effect measures, LOR, LRR, and RD, and investigated their quality by simulation. For comparison, we also considered two approximations to the corresponding null distributions of $Q_{IV}$: the standard chi-square approximation and the improved approximations by Kulinskaya et al. for RD [18] and Kulinskaya and Dollinger for LOR [11].

The four new approximations are based on the Farebrother approximation, which works well under normality, and the two-moment gamma approximation. For each of these approximations, we investigated two approaches to estimation of $p_{iT}$ (which is used in the calculation of the second and fourth central moments of an effect measure): “naïve” estimation of $p_{iT}$ from $X_{iT}$ and $n_{iT}$ and “model-based” estimation, which uses the fixed-effects meta-analysis estimate of the overall effect to obtain $\hat{p}_{iT}$ from $\hat{p}_{iC}$. Overall, the 2-moment gamma approximation proved better for small sample sizes and/or probabilities, where the distributions of the binary effect measures are far from normal. However, the findings are not straightforward and very much depend on the particular effect measures and configurations. The risk difference presents the best-case scenario: the F SSW model approximation provides very good results for very small sample sizes, $n = 20$ and $\bar{n} = 30$.

Including previous work [7, 8], we now have extensive results, for five common measures of effect (MD, SMD, LOR, LRR, and RD), on the performance of $Q_F$, on accuracy of approximations to its distributions, and on comparisons with $Q_{IV}$. The details confirm that the features for binary data differ substantially from those for, say, normal means. Also, the present paper demonstrates that the *Q* statistics behave very differently for risk differences and log-odds-ratios. Thus, each type of data requires a specific practical solution. This paper and our previous papers are necessary steps in that direction. They should point the way to further improvements.

An additional advantage of the sample-size-weighted methods, including $Q_F$, is that they would be more robust than the IV methods, to publication bias. Publication bias may arise from a negative association between sample sizes and effect sizes in meta-analyzed studies. SSW methods downweight these inflated results from the small studies. Inverse-variance weights are also inversely proportional to sample sizes, but for binary data, they also depend on the probabilities in the control arm and the effects. Therefore, their behavior under small-sample bias may be quite erratic.

One could consider assessing heterogeneity by estimating the parameter for the between-study variance in a mixed-effects logistic regression model. That approach, however, would assume likelihoods that $Q_F$ does not need, and results in [28] and [29] show that it does not work well.

## Conclusions

For LOR or LRR, dependence on $p_{iC}$ and the effect value makes choice of an approximation for the null distribution of *Q* rather challenging, though the inadequate performance of the standard $\chi ^2$ approximation is quite a universal conclusion. The improved approximations by Kulinskaya et al. [18] and Kulinskaya and Dollinger [11] to the null distribution of $Q_{IV}$ work better than the $\chi ^2$ approximation for $n \ge 100$, though they may encounter some convergence issues for small sample sizes and/or probabilities. Instead, we recommend using a test of heterogeneity based on $Q_F$ and provide practical guidelines for choosing an appropriate test at the .05 level for all three effect measures.

In further work, we intend to explore related methods of estimating the heterogeneity variance $\tau ^2$ based on $Q_F$.

## Supplementary Information

**Additional file 1.**

## Data Availability

Our full simulation results are available as an arXiv e-print (arXiv:2206.08907v2). The user-friendly R programs implementing all the investigated *Q* tests for heterogeneity in meta-analysis of LOR, LRR, and RD are available at https://osf.io/yqgsk. These also includes examples of meta-analysis of the data from Stead et al. [26]. Please contact E. Kulinskaya with any queries.

## Abbreviations

2M SSW naïve: Two-moment Gamma SSW approximation based on “naïve” estimation of $p_{iT}$ from $X_{iT}$ and $n_{iT}$
2M SSW model: Two-moment Gamma SSW approximation based on “model-based” estimation using the fixed-effect meta-analysis estimate of the overall effect to obtain $\hat{p}_{iT}$ from $\hat{p}_{iC}$
ChiSq: Chi-square approximation
F SSW naïve: Farebrother SSW approximation based on “naïve” estimation of $p_{iT}$ from $X_{iT}$ and $n_{iT}$
F SSW model: Farebrother SSW approximation based on “model-based” estimation using the fixed-effect meta-analysis estimate of the overall effect to obtain $\hat{p}_{iT}$ from $\hat{p}_{iC}$
IV: Inverse-variance (weights)
KD: Approximation of Kulinskaya and Dollinger [11]
KDB: Approximation of Kulinskaya et al. [18]
LOR: Log-odds-ratio
LRR: Log-risk-ratio
MD: Mean difference
MP: Mandel-Paule estimator
MSE: Mean squared error
OR: Odds-ratio
RD: Risk difference
REM: Random-effects model
RR: Risk ratio (relative risk)
SMD: Standardized mean difference
SSW: Effective-sample-size weights
