# Supplementary material for: On the Q statistic with constant weights in meta-analysis of binary outcomes
Source: BMC Med Res Methodol. 2023 Jun 21;23:146. doi: 10.1186/s12874-023-01939-z (PMC10286409; doi:10.1186/s12874-023-01939-z)
Supplement: Supplementary file 1 — Additional file 1. [file 12874_2023_1939_MOESM1_ESM.pdf]

Supplementary Materials

for

Elena Kulinskaya and David C. Hoaglin

On the Q statistic with constant weights in meta-analysis of binary outcomes

A.1 Calculation of the conditional central moments of $\hat{\theta}$

Let $\hat{\theta} = h(\hat{p}_T) - h(\hat{p}_C)$ be an unbiased estimator of $\theta = h(p_T) - h(p_C)$. As in Section ??, we write $\Theta = \hat{\theta} - \theta$. Since $\hat{p}_C$ and $\hat{p}_T$ are independent, the conditional central moments of $\hat{\theta}$ (given the values of n and p) are as follows:

$$
\begin{aligned}
M_1^c = \mathrm{E}[\Theta] &= 0 \\
M_2^c = \mathrm{E}[\Theta^2] &= M_2[h(\hat{p}_T)] + M_2[h(\hat{p}_C)] \\
M_3^c = \mathrm{E}[\Theta^3] &= M_3[h(\hat{p}_T)] - M_3[h(\hat{p}_C)] \\
M_4^c = \mathrm{E}[\Theta^4] &= M_4[h(\hat{p}_T)] + 6M_2[h(\hat{p}_T)]M_2[h(\hat{p}_C)] + M_4[h(\hat{p}_C)] \\
M_5^c = \mathrm{E}[\Theta^5] &= M_5[h(\hat{p}_T)] + 10M_3[h(\hat{p}_T)]M_2[h(\hat{p}_C)] - 10M_2[h(\hat{p}_T)]M_3[h(\hat{p}_C)] - M_5[h(\hat{p}_C)] \\
M_6^c = \mathrm{E}[\Theta^6] &= M_6[h(\hat{p}_T)] + 15M_4[h(\hat{p}_T)]M_2[h(\hat{p}_C)] - 20M_3[h(\hat{p}_T)]M_3[h(\hat{p}_C)] \\
&\quad +15M_2[h(\hat{p}_T)]M_4[h(\hat{p}_C)] + M_6[h(\hat{p}_C)]
\end{aligned}
\tag{A.1}
$$

A.2 Conditional central moments for RD

This section gives the necessary formulas for the conditional central moments of the risk difference $\hat{\Delta} = \hat{p}_T - \hat{p}_C$.

Following Kulinskaya et al. (2011), to find the conditional central moments for RD (if $\tilde{p} = X/n$ is used), we substitute the following binomial central moments $M_k[\hat{p}] =$

$\mathrm{E}[(\hat{p}-p)^k]$ into Equation (A.1):

$$\begin{aligned}
M_1[\tilde{p}] &= 0 \\
M_2[\tilde{p}] &= \frac{p(1-p)}{n} \\
M_3[\tilde{p}] &= \frac{(1-2p)p(1-p)}{n^2} \\
M_4[\tilde{p}] &= \frac{3p^2(1-p)^2}{n^2} + \frac{p(1-p)(1-6p(1-p))}{n^3} \\
M_5[\tilde{p}] &= \frac{10(1-2p)p^2(1-p)^2}{n^3} + \frac{(1-2p)p(1-p)(1-12p(1-p))}{n^4} \\
M_6[\tilde{p}] &= \frac{15p^3(1-p)^3}{n^3} + \frac{5p^2(1-p)^2(5-26p(1-p))}{n^4} \\
&\quad + \frac{p(1-p)(1-30p(1-p)+120p^2(1-p)^2)}{n^5}
\end{aligned}$$

A.3 Conditional central moments for estimated logit or log of a binomial probability

These moments for any function $h(X)$ can be calculated exactly, as suggested in Kulinskaya and Dollinger (2015), by calculating, for given values of p and n, the binomial probabilities $B(x;p,n)$ for $x=0,\ldots,n$, and then calculating the expected value $\mathrm{E}(h(X)^k|p,n) = \sum_x B(x;p,n)h^k(x)$. In practice, they are estimated by plugging in a relevant estimate of p.

A.4 Stead et al. (2013) data on smoking cessation

Study	X_T	n_T	X_C	n_C
Schnoll 2003	27	203	28	206
Vetter 1990	34	237	20	234
Higashi 1995	53	468	35	489
Russell 1979	34	1031	8	1107
Slama 1990	1	104	1	106
Janz 1987	26	144	12	106
Demers 1990	15	292	5	292
Stewart 1982	11	504	4	187
McDowell 1985	12	85	11	78
Wilson 1990	43	577	17	532
Russell 1983	43	740	35	637
Jamrozik 1984	77	512	58	549
Page 1986	8	114	5	68
Slama 1995	42	2199	5	929
Nebot 1989	11	208	5	216
Betson 1997	14	443	13	422
Porter 1972	5	101	4	90
Unrod 2007	28	237	18	228

Table A.1: Stead et al. (2013) data on the use of physician advice for smoking cessation

A.5 Additional figures

Additional relative error plots vs upper tail area and the plots of empirical levels at 5% significance level of the heterogeneity tests for LOR, LRR and RD.

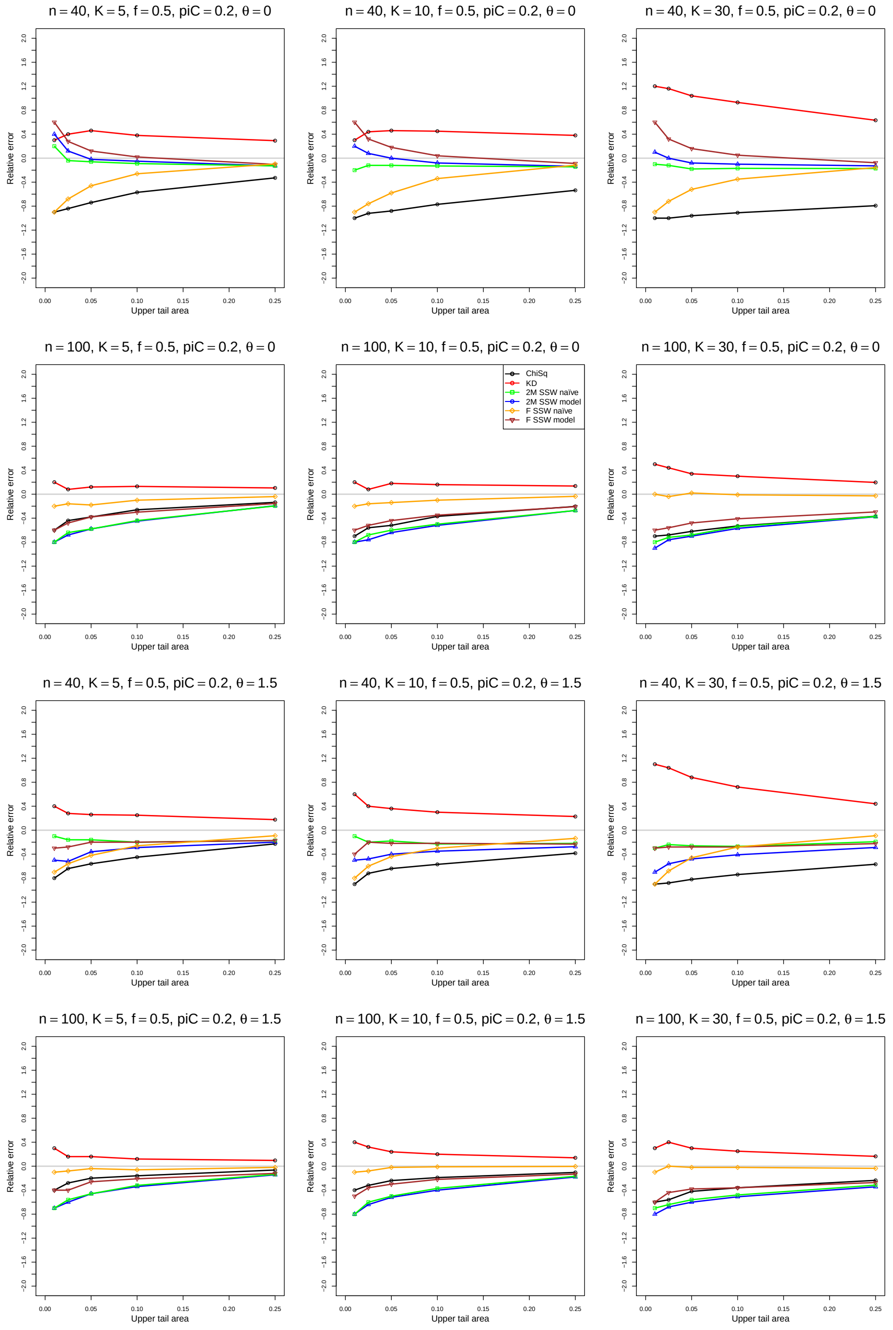

Figure A.1: Relative error in the level of the test for heterogeneity of log-odds-ratio, vs upper tail area, for four approximations to the null distribution of Q_F and two approximations to the null distribution of Q_{IV}, when $p_{iC} = .2$. The rows correspond to the combinations of $\theta = 0$ and $\theta = 1.5$ with $n = 40$ and $n = 100$.

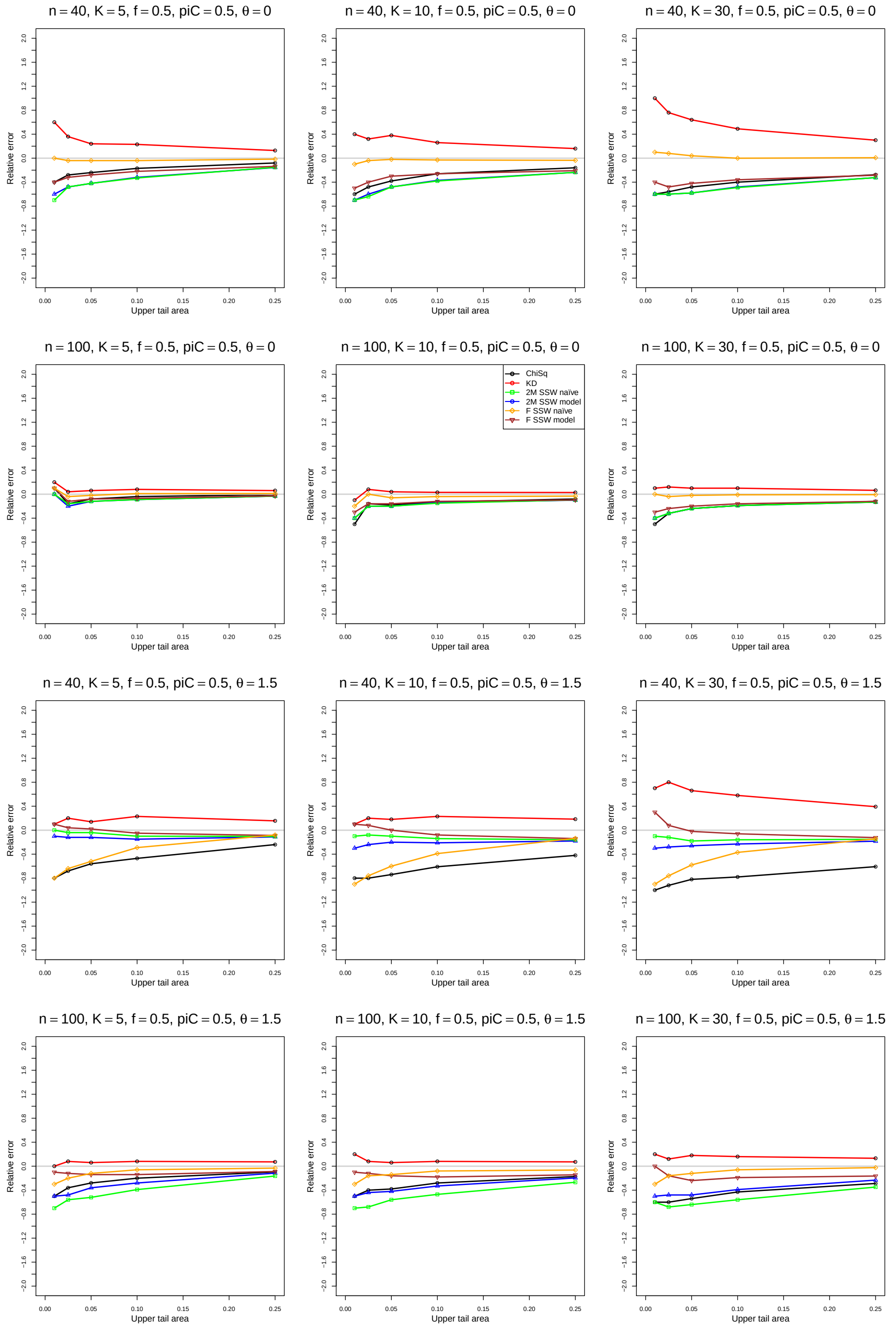

Figure A.2: Relative error in the level of the test for heterogeneity of log-odds-ratio, vs upper tail area, for four approximations to the null distribution of Q_F and two approximations to the null distribution of Q_{IV}, when $p_{iC} = .5$. The rows correspond to the combinations of $\theta = 0$ and $\theta = 1.5$ with $n = 40$ and $n = 100$.

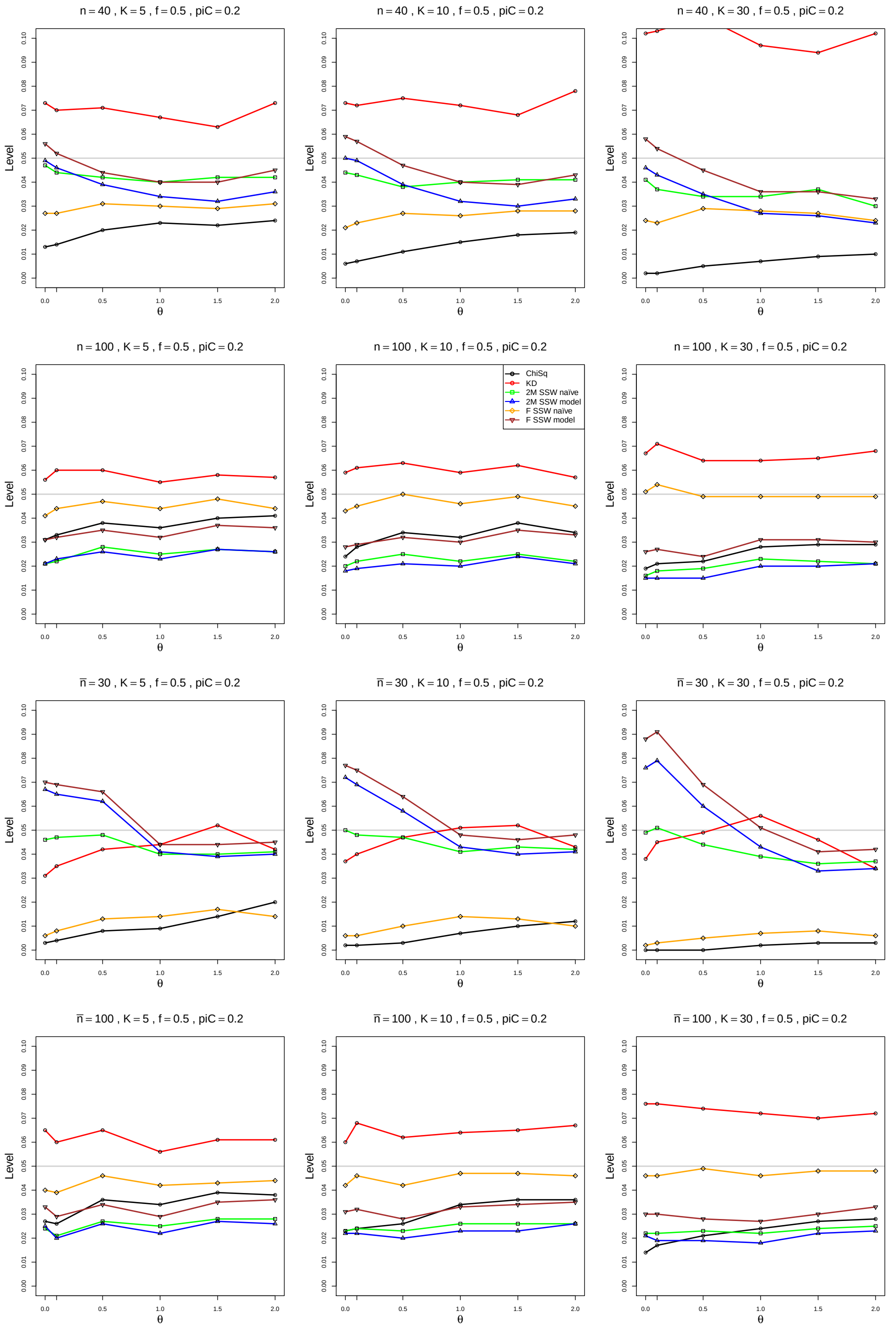

Figure A.3: Empirical levels at nominal level of significance .05, vs θ, when $p_{iC} = .2$, for four approximations to the distribution of Q_F for log-odds-ratio and for two approximations to the null distribution of Q_{IV}. First two rows: equal sample sizes $n = 40$ and 100; second two rows: unequal sample sizes, $\bar{n} = 30$ and 100

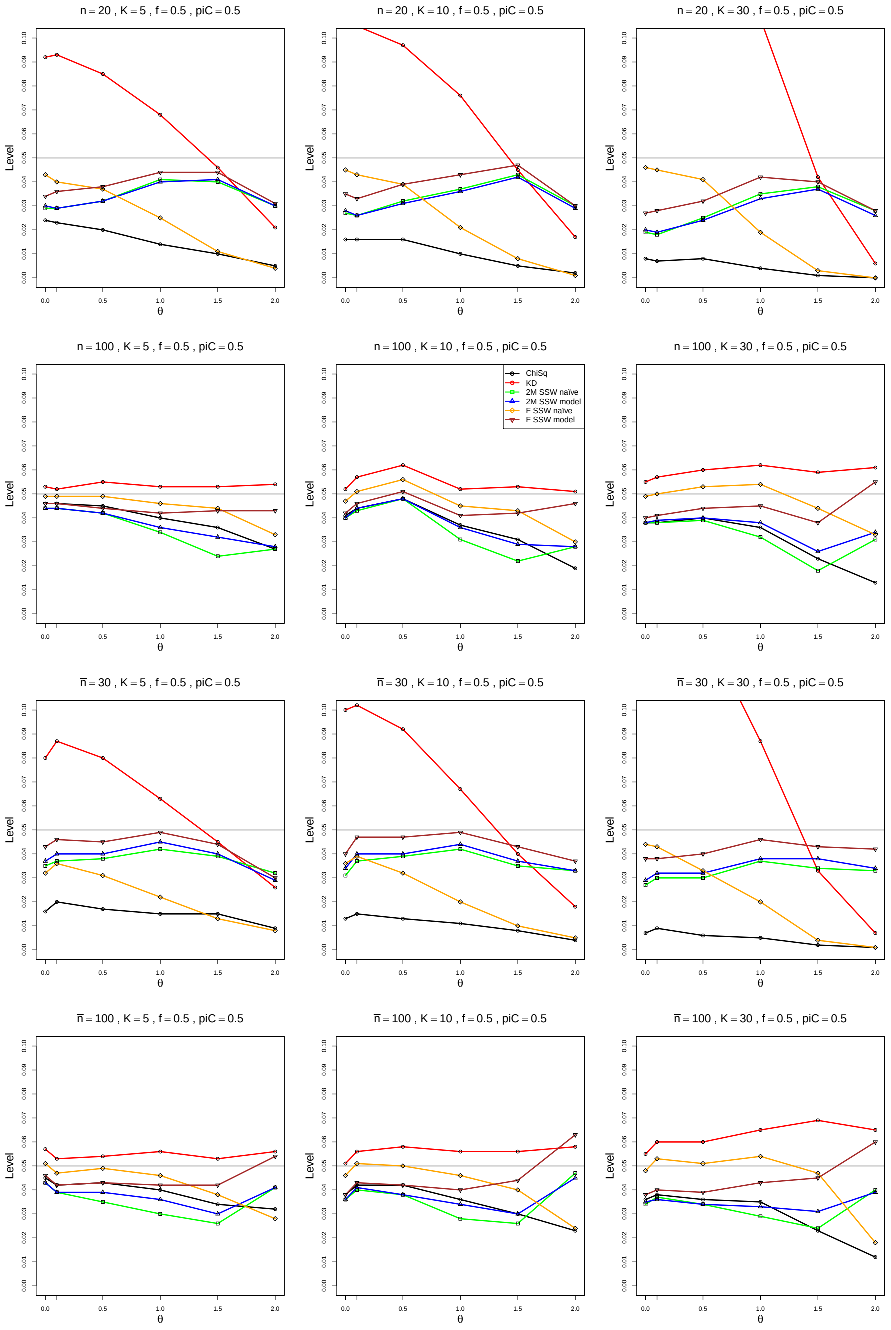

Figure A.4: Empirical levels at nominal level of significance .05, vs θ, when $p_{iC} = .5$, for four approximations to the distribution of Q_F for log-odds-ratio and for two approximations to the null distribution of Q_{IV}. First two rows: equal sample sizes $n = 20$ and 100; second two rows: unequal sample sizes, $\bar{n} = 30$ and 100

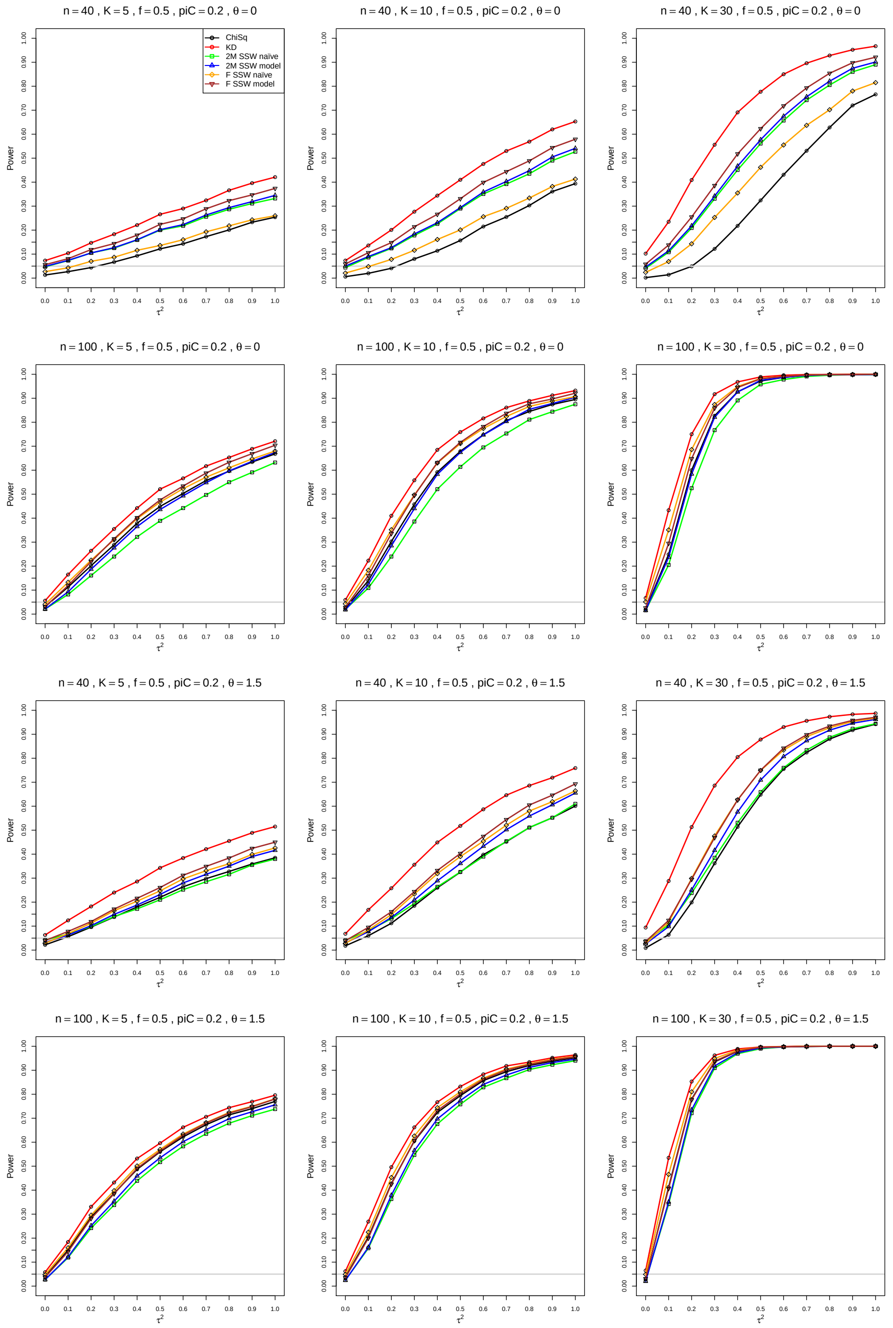

Figure A.5: Empirical power at nominal level $\alpha = .05$ vs τ^2, for four approximations to the distribution of Q_F for log-odds-ratio and for two approximations to the null distribution of Q_{IV}, when $p_{iC} = .2$, $f = .5$, equal sample sizes. The rows correspond to the combinations of $\theta = 0$ and $\theta = 1.5$ with $n = 40$ and $n = 100$.

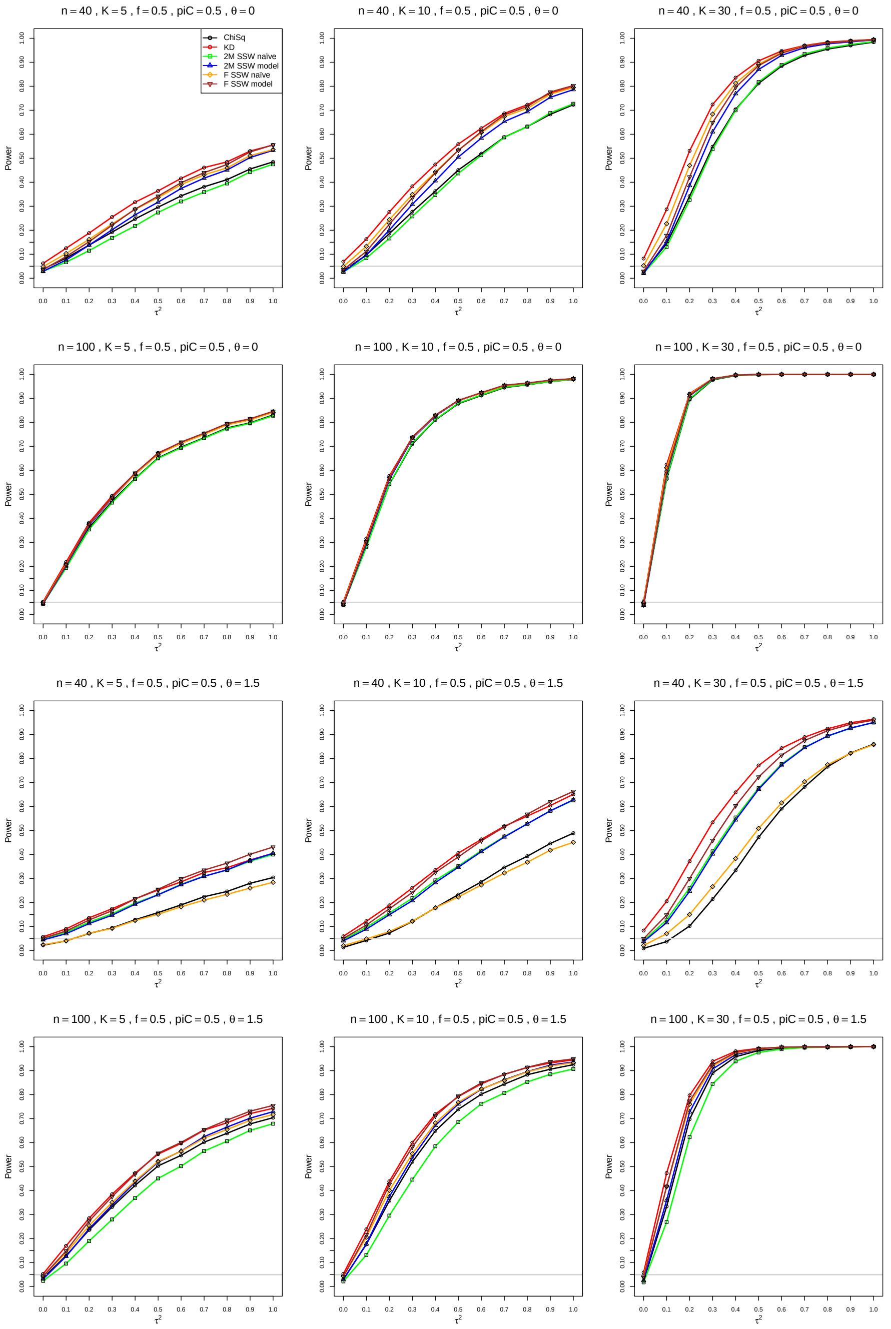

Figure A.6: Empirical power at nominal level $\alpha = .05$ vs τ^2, for four approximations to the distribution of Q_F for log-odds-ratio and for two approximations to the null distribution of Q_{IV}, when $p_{iC} = .5$, $f = .5$, equal sample sizes. The rows correspond to the combinations of $\theta = 0$ and $\theta = 1.5$ with $n = 40$ and $n = 100$.

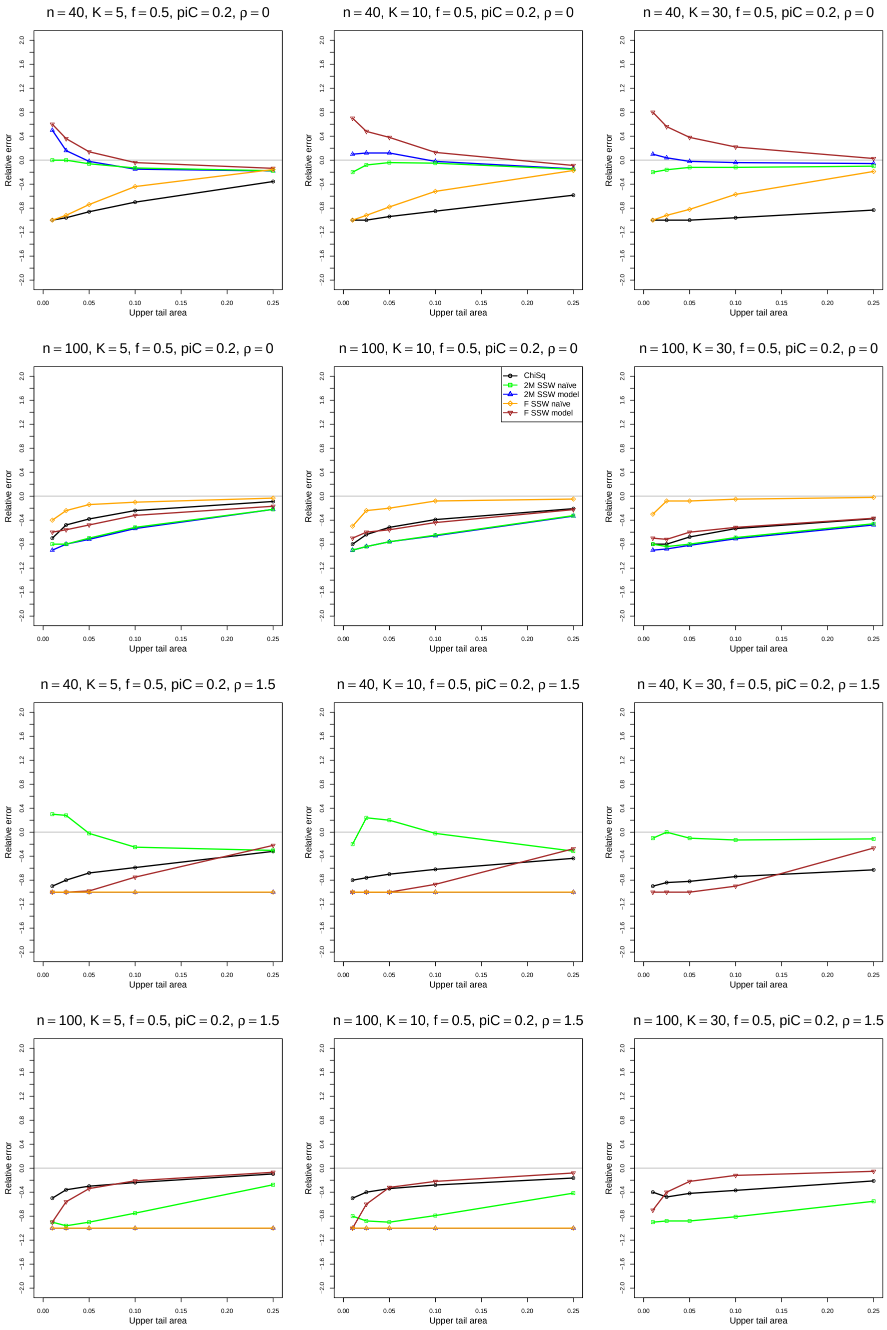

Figure A.7: Relative error in the level of the test for heterogeneity of log-relative-risk, vs upper tail area, for four approximations to the null distribution of Q_F and the chi-square approximation to the null distribution of Q_{IV}, when $p_{iC} = .2$. The rows correspond to the combinations of $\rho = 0$ and $\rho = 1.5$ with $n = 40$ and $n = 100$.

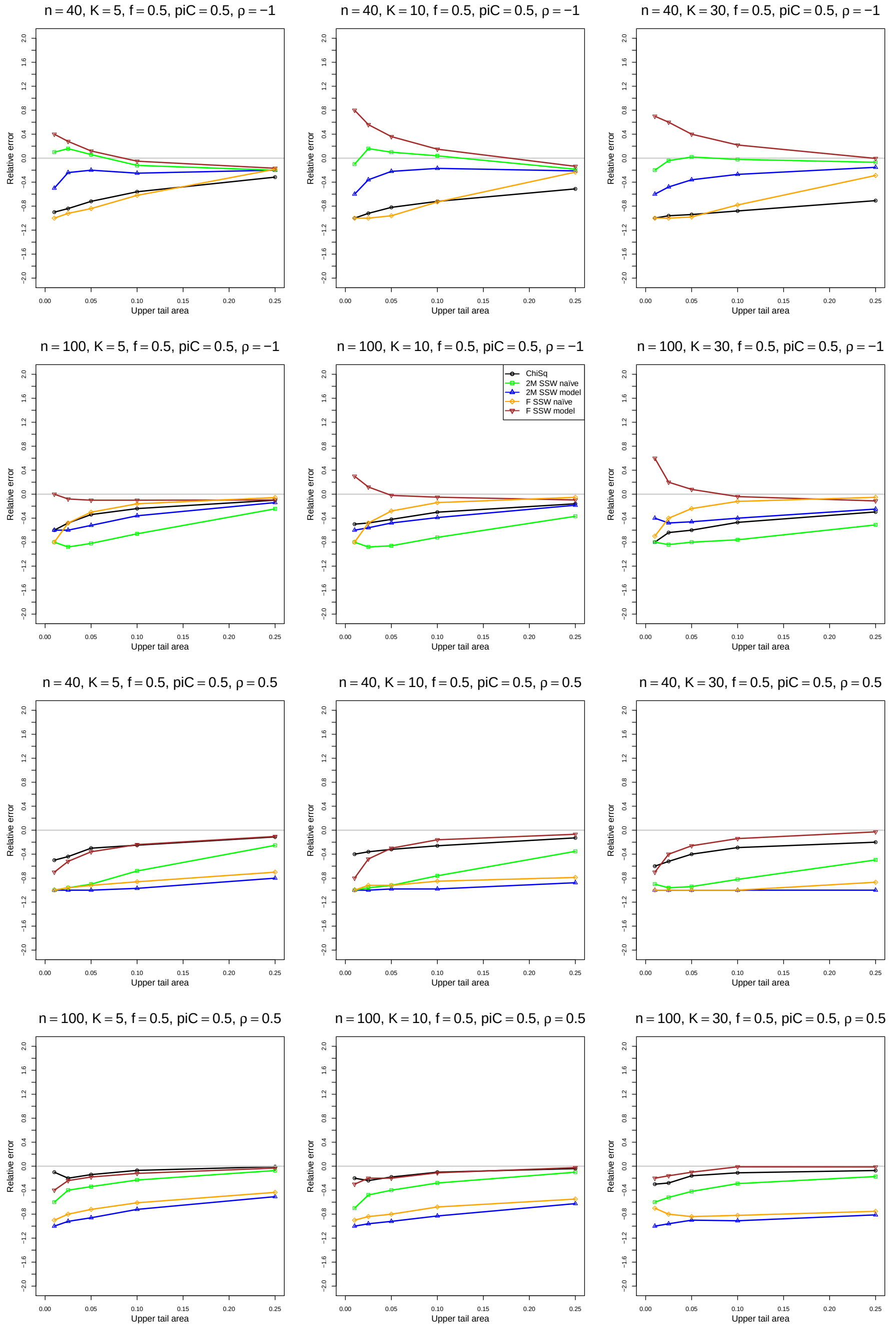

Figure A.8: Relative error in the level of the test for heterogeneity of log-relative-risk, vs upper tail area, for four approximations to the null distribution of Q_F and the chi-square approximation to the null distribution of Q_{IV}, when $p_{iC} = .5$. The rows correspond to the combinations of $\rho = -1$ and $\rho = 0.5$ with $n = 40$ and $n = 100$.

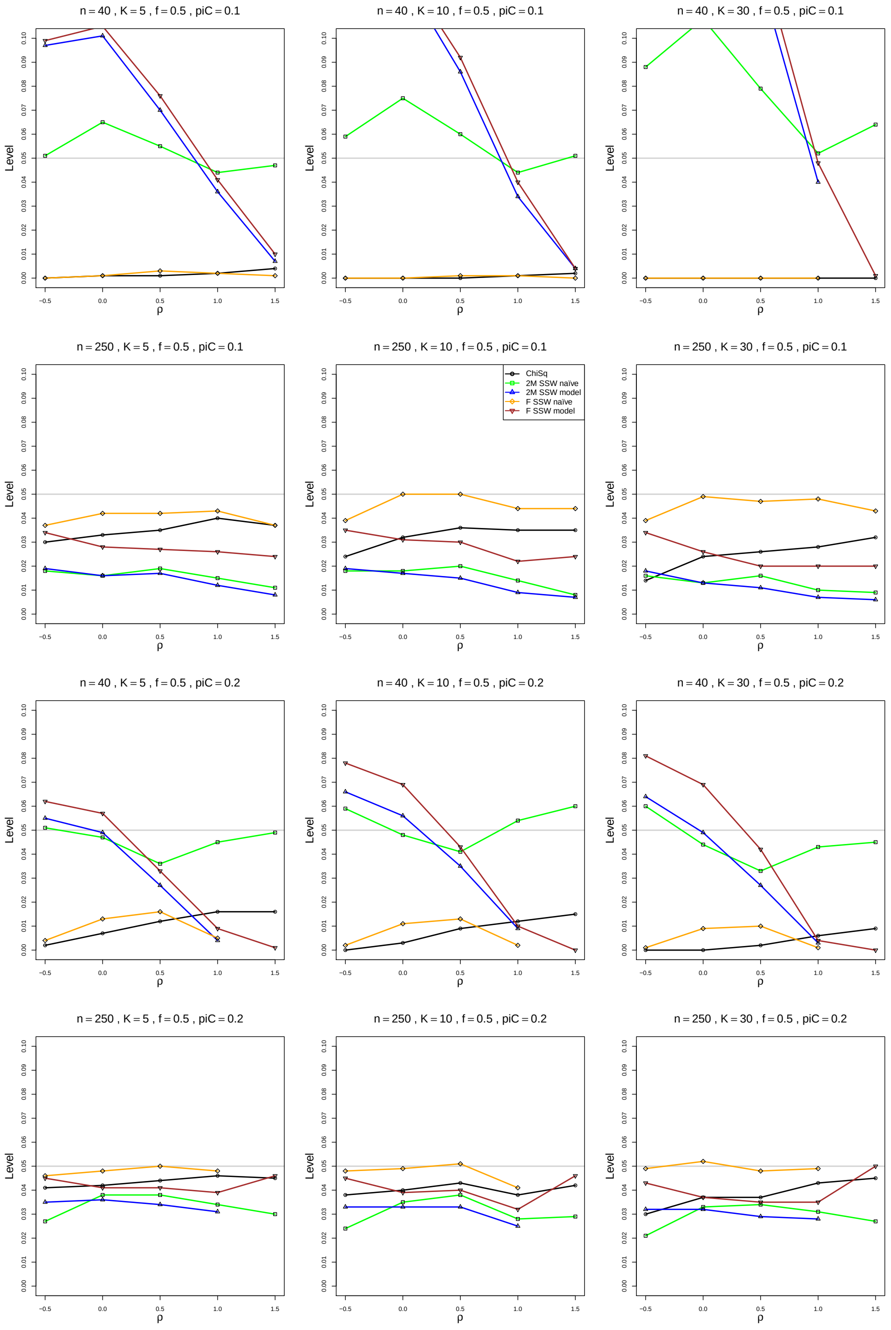

Figure A.9: Empirical levels at nominal level of significance .05, vs ρ, for four approximations to the null distribution of Q_F for log-relative-risk and for the standard chi-square approximation to the null distribution of Q_{IV}, for equal sample sizes, $n = 40$ and 250. First two rows: $p_{iC} = .1$; second two rows: $p_{iC} = .2$.

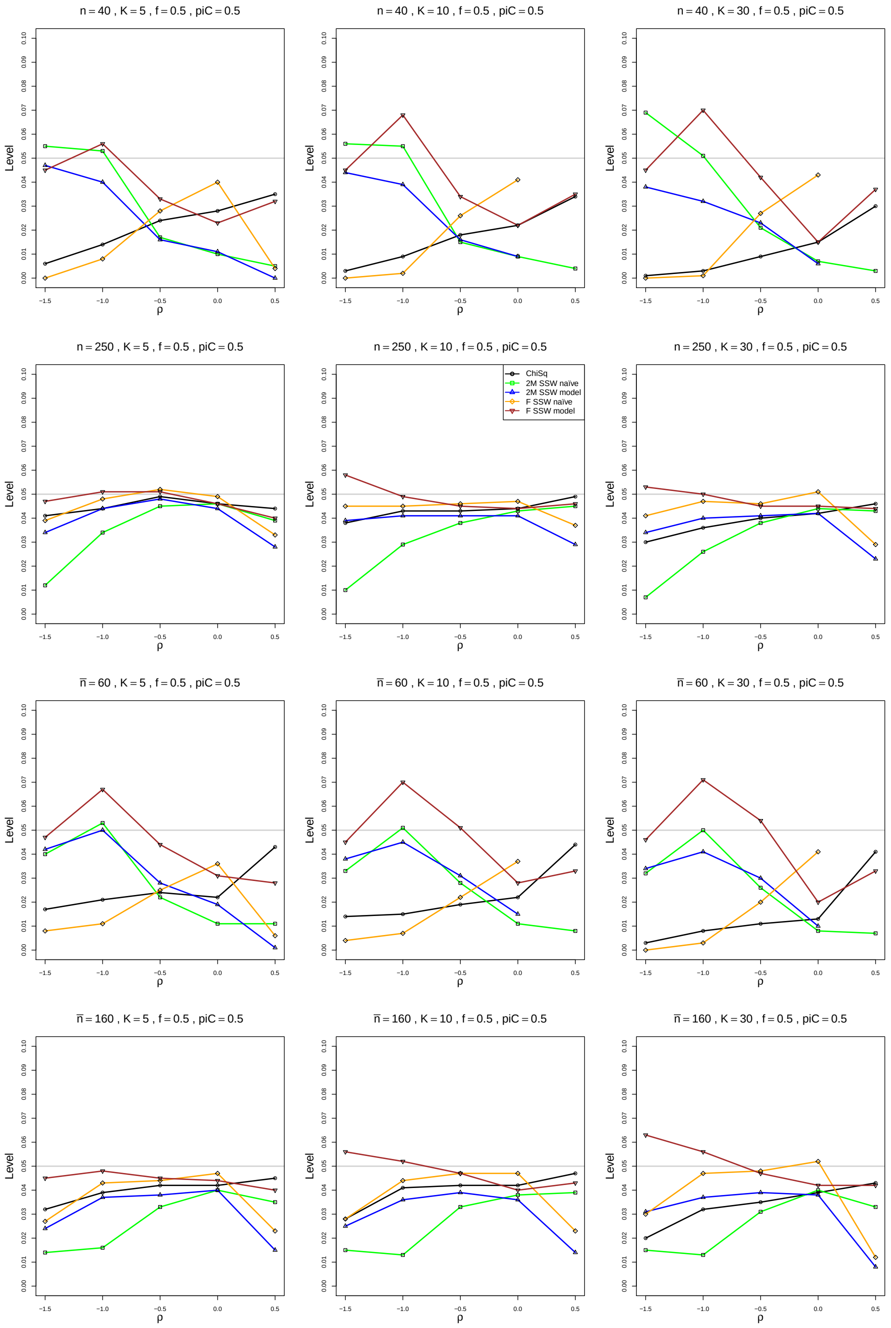

Figure A.10: Empirical levels at nominal level of significance .05, vs ρ, when $p_{iC} = .5$, for four approximations to the null distribution of Q_F for log-relative-risk and for the standard chi-square approximation to Q_{IV}. First two rows: equal sample sizes, $n = 40$ and 250; second two rows: unequal sample sizes, $\bar{n} = 60$ and 160.

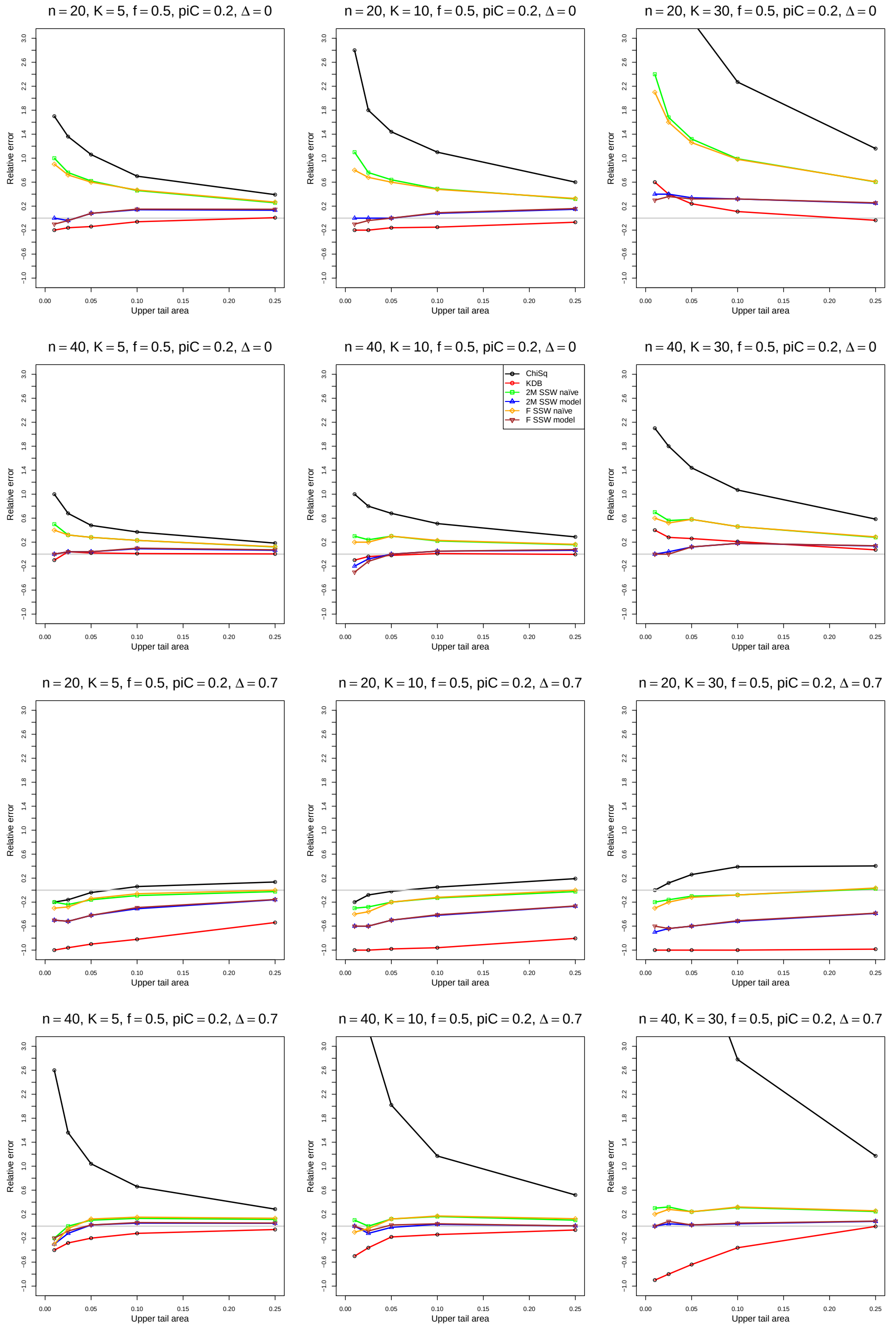

Figure A.11: Relative error in the level of the test for heterogeneity of risk difference, vs upper tail area, for four approximations to the null distribution of Q_F and two approximations to the null distribution of Q_{IV}, when $p_{iC} = .2$. The rows correspond to the combinations of $\Delta = 0$ and $\Delta = 0.70$ with $n = 20$ and $n = 40$.

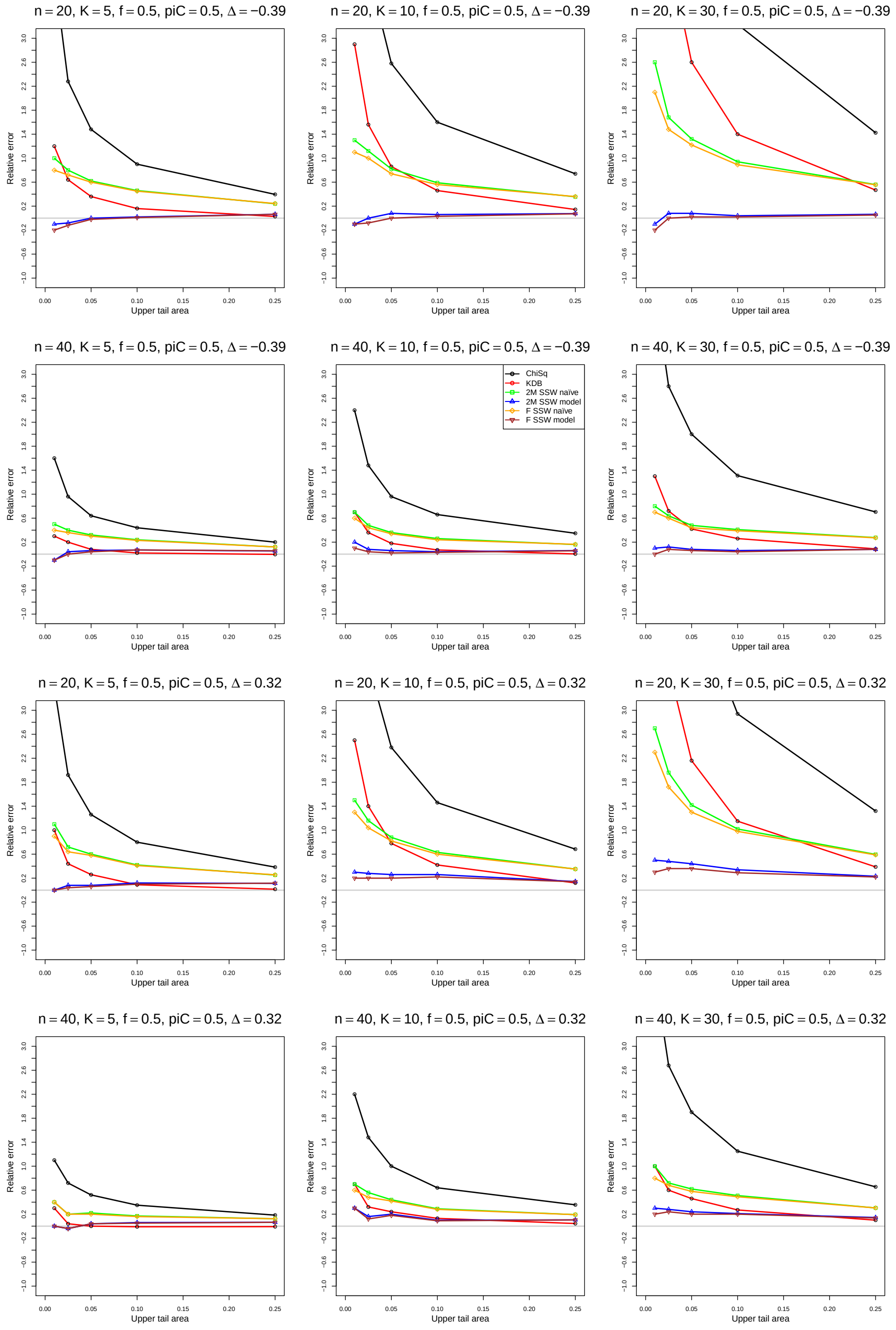

Figure A.12: Relative error in the level of the test for heterogeneity of risk difference, vs upper tail area, for four approximations to the null distribution of Q_F and two approximations to the null distribution of Q_{IV}, when $p_{iC} = .5$. The rows correspond to the combinations of $\Delta = -0.39$ and $\Delta = 0.32$ with $n = 20$ and $n = 40$.

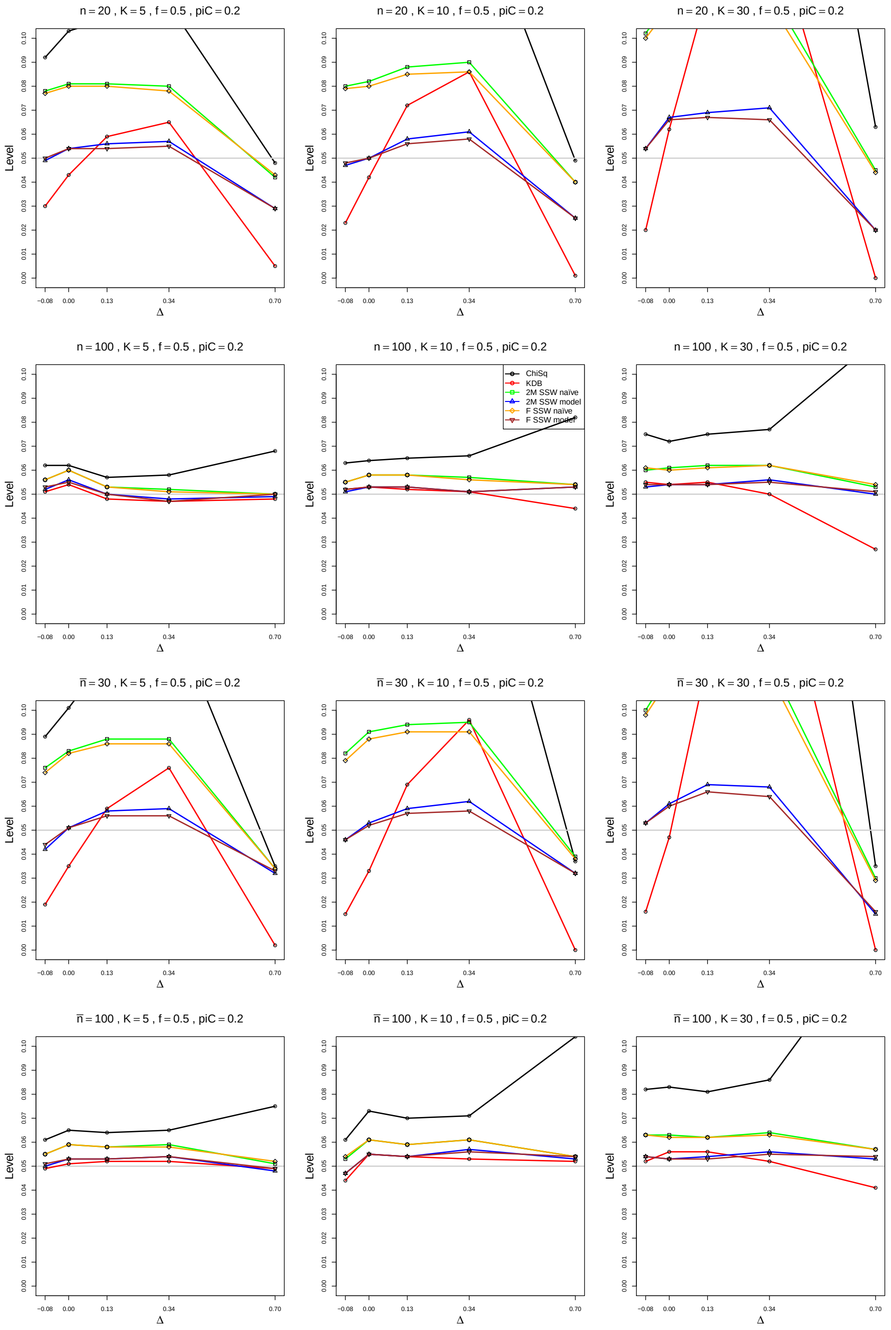

Figure A.13: Empirical levels at nominal level of significance .05, vs Δ, when $p_{iC} = .2$, for four approximations to the null distribution of Q_F and two approximations to the null distribution of Q_{IV} for risk difference. First two rows: equal sample sizes $n = 40$ and 100; second two rows: unequal sample sizes, $\bar{n} = 30$ and 100.

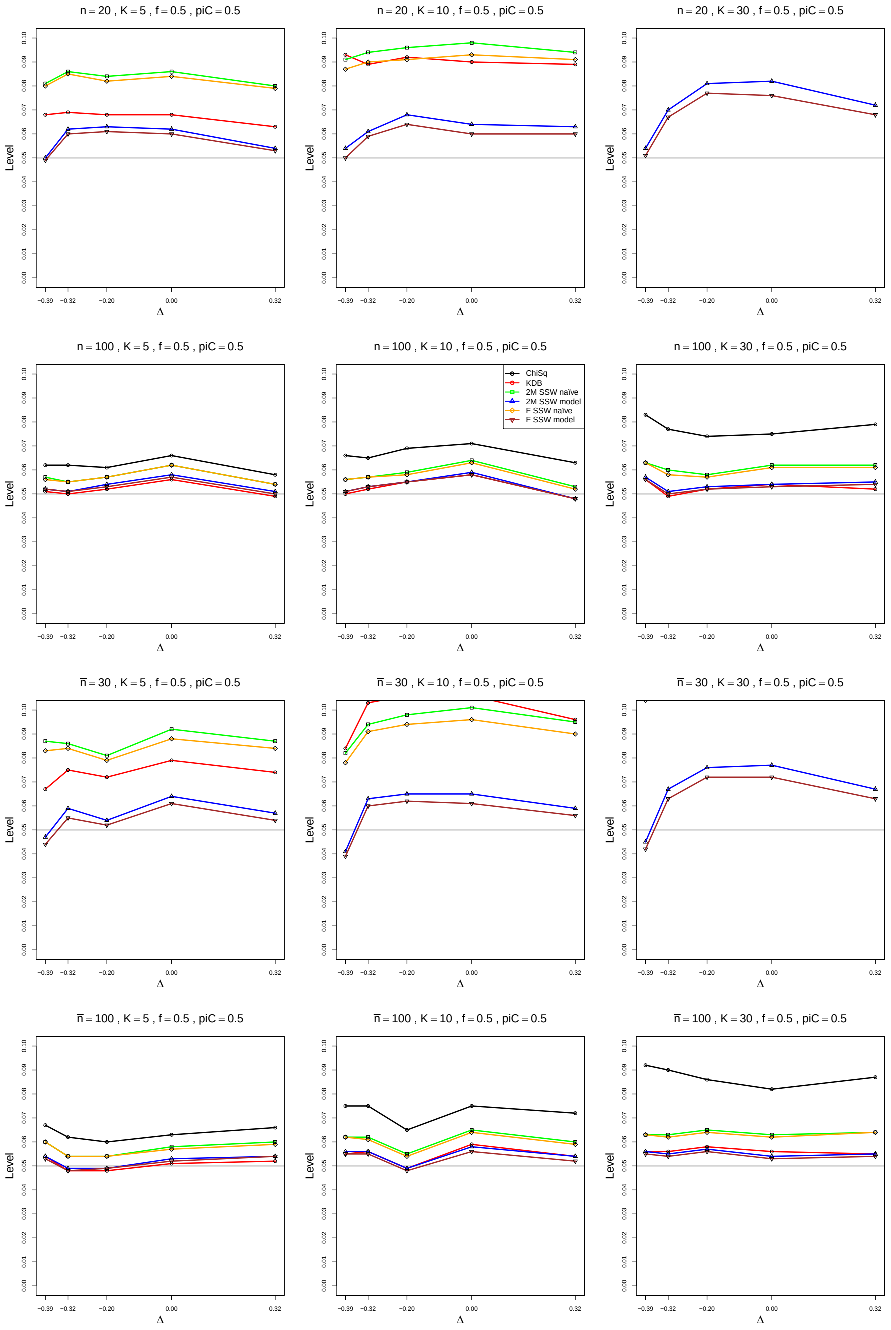

Figure A.14: Empirical levels at nominal level of significance .05, vs Δ, when $p_{iC} = .5$, for four approximations to the null distribution of Q_F and two approximations to the null distribution of Q_{IV} for risk difference. First two rows: equal sample sizes $n = 40$ and 100; second two rows: unequal sample sizes, $\bar{n} = 30$ and 100.
